# An Efficient Descriptor for Tubular LDPE Melt Strength via Physics-Guided Learning and Diffusion-Based Inverse Design

**DOI:** 10.3390/polym18192421

**Published:** 2026-10-04

**Authors:** Yifei Wang, Guogang Liu, Lin Liu, Yue Yu, Peiqian Yu, Linge Ma, Linlin Wei, Zhe Zhou, Linfeng Chen, Delin Yuan, Rongjuan Cong

**Affiliations:** National Institute of Clean-and-Low-Carbon Energy, Beijing 102209, China

**Keywords:** polyolefin characterization, low-density polyethylene (LDPE), physics-guided machine learning, structure–property relationship, data-driven polyolefin optimization, melt strength, GPC, multi-detector GPC (MD-GPC), molecular architecture, inverse molecular design, diffusion model, branching index (gpcBr), tubular LDPE

## Abstract

Melt strength (MS) is a key parameter governing the processability and application of low-density polyethylene (LDPE), dictated by a complex interplay of molecular weight distribution (MWD), long-chain branching (LCB), and high-molecular-weight content—features poorly captured by conventional metrics like weight average molecular weight (Mw). This paper addresses the challenge by developing a physics-guided machine learning framework that constructs a list of molecular descriptors directly from full-spectrum MWD data using modern multi-detector high-temperature gel permeation chromatography (MD-GPC), explicitly aligned with the polymer entanglement fundamentals underlying melt strength. The descriptor enables accurate prediction and interpretable disentanglement of topological contributions. The paper further introduced a diffusion-based generative model for inverse design: given a target melt strength, the model proposed candidate molecular architectures and validated them through internal consistency with the learned structure–property relationships, offering a rational and data-driven strategy for the precise engineering of LDPE molecular structures, thus providing considerable flexibility for downstream engineering decisions. Practitioners may further refine or screen these candidates in light of real-world factors—including polymerization processing constraints, cost considerations for resin selection and blending, and manufacturing operation windows—thereby tailoring LDPE molecular structures/products to specific application requirements with high precision.

## 1. Introduction

Low-density polyethylene (LDPE), a cornerstone of the global polyolefin industry, is extensively utilized in packaging, automotive, telecommunications, and medical applications owing to its exceptional melt strength (MS) [1]. Melt strength, defined as the maximum extensional force (in units of Newton, N) that a polymer melt can sustain before rupture, governs the stability of extensional flow processes. In film blowing, for example, insufficient MS leads to bubble instability, non-uniform film thickness, and ultimately compromised mechanical performance of the final product [2]. Designing LDPE resins to meet a target MS specification has therefore remained a central yet formidable challenge across polyolefin research and development.

The MS of LDPE is intrinsically governed by its heterogeneous molecular architecture, which is collectively shaped by polymerization conditions—including pressure, temperature, initiators and their injection sites, and reactor configuration. The relevant structural descriptors are inherently multidimensional, encompassing weight-average molecular weight (Mw), molecular weight distribution (MWD), short-chain branching (SCB) generated via back-biting mechanisms during free-radical polymerization (typically 5–30 methyl groups per 1000 total carbon atoms, CH_3_/1000 C), and diverse long-chain branching (LCB) topologies (e.g., comb-like or star-like architectures) and content. At a molecular level, MS is fundamentally governed by chain entanglement and extensional strand dynamics under processing-relevant deformation [3,4]. LDPE samples sharing an identical Mw can exhibit markedly divergent MS values due to the differences in higher-order structural parameters such as polydispersity index (PDI) and LCB characteristics. These features, which govern entanglement-network formation and strain-hardening behavior, cannot be fully or precisely captured by Mw alone [5]. Data-driven modeling offers transformative potential for establishing robust structure–property relationships amid such interdependent parameters via physics-guided interpretable feature engineering [6]; however, its success hinges on the availability of high-quality, reproducible datasets [7]—a longstanding bottleneck in LDPE research arising from the inherent difficulties of both molecular structural characterization and melt strength measurement.

^13^C NMR is another widely used technique to quantify LCB longer than approximately C8. For branches having C18 vs. C38 for example, there is no difference in the corresponding chemical shifts [8]. Consequently, LCB measurements derived from NMR may differ significantly from those obtained via 3D-GPC for some polyolefin materials. This discrepancy between techniques underscores the need for future studies to incorporate advanced experimental approaches—such as molecular structural fractionation or specialized flow through NMR measurement—capable of selectively and accurately quantifying LCB to further refine melt strength predictions. Accurate quantification of LCB by multi-detector high-temperature gel permeation chromatography (MD-GPC) requires identifying suitable reference standards and models to calculate the reduction in molecular size relative to linear chains of identical SCB content (and type) and molecular weight [9,10]. Fundamentally, this requirement remains unmet for LDPE owing to its structural complexity, including heterogeneous SCB types, SCB contents and varied LCB topologies [11]. Recent advances in modern MD-GPC instrumentation—incorporating high signal-to-noise multi-angle light scattering (MALS), viscometry, and filter-based infrared (IR) detectors [12,13] equipped with an MCT (Mercury Cadmium Telluride)-cooled infrared detector for substantially improved signal-to-noise ratios—have enabled comprehensive quantification of backbone Mw, absolute Mw (Mw(LS)), MWD, intrinsic viscosity of the bulk (IV), and SCB distribution across the MWD spectrum. Notably, MALS provides at least twice the signal-to-noise ratio of even the most sensitive two capillary viscometers, markedly enhancing measurement fidelity [14]. Coupled with optimized sample preparation via automated samplers [15], rigorous calibration protocols, enhanced detector sensitivity, and statistical quality control [16], these advances have reduced relative standard deviations to ≤1.8% for key parameters such as Mw, IV, and Mw(LS). This level of analytical reproducibility establishes a robust foundation for leveraging full-spectrum MWD data in deployable machine learning models for real-world polymer design applications.

Data-driven computational methods have recently revealed structure–property correlations previously obscured by conventional manual analysis [17,18]. Machine learning (ML) models, including random forests and neural networks, excel at integrating weak signals to decode complex molecular architectures, demonstrating outstanding accuracy in predicting the chemical composition of recycled post-consumer plastics relative to traditional regression approaches. However, unconstrained AI models carry inherent risks, such as generating physically implausible predictions. Physics-constrained AI addresses this limitation by integrating fundamental physical and structural principles—such as polymer entanglement theory and branching thermodynamics—directly into the modeling process, thereby disentangling the contributions of individual parameters from their interactive effects and enhancing both predictive reliability and interpretability.

Building upon these advances, the present work combines state-of-the-art MD-GPC characterization protocols with our recently standardized methodology for extracting MS data from capillary rheometry (Rheotens) measurements. This study provides a detailed and quantitative approach to differentiate tubular LDPE grades that meet very similar target density and melt index specifications but exhibit noticeable performance behaviors on actual fabrication lines at customer facilities. Leveraging this well-curated dataset, we investigate the quantitative correlations between MS and multidimensional molecular structural parameters, and develop a physics-constrained data-driven framework that overcomes the limitations of conventional empirical correlations, offering a scalable and interpretable model for MS prediction. Furthermore, by capitalizing on the probabilistic generative nature of diffusion models, we enable the exploration of diverse molecular architectures conditioned on target MS properties. This inverse design capability facilitates the predictive development of novel LDPE materials with tailored rheological performance, paving the way for accelerated, data-driven advancements in polymer engineering.

## 2. Materials and Methods

Fifteen low-density polyethylene (LDPE) samples (Table S1, Supplementary Materials) produced in tubular process by various manufacturers located in China were used in this study. Data analysis was performed using Python (v3.10).

### 2.1. Molecular Weight Distribution (MWD)

MWD was characterized by high-temperature GPC coupled with a filter-based infrared detector (IR6), a four-capillary viscometer, and an 8-angle light scattering (MALS) detector (GPC-IR, PolymerChar, Paterna, Spain; MALS, Waters Inc., Milford, MA, USA). Separation was achieved on three PLgel Olexis columns (13 µm, Agilent Technologies, Inc., Santa Clara, CA, USA) at 150 °C using 1,2,4-trichlorobenzene (TCB) containing 200 ppm BHT as the mobile phase. Sample concentration was 2.0 mg/mL. Polyethylene equivalent (also referred as backbone) MWD were determined via universal calibration with 16 polystyrene standards (PolymerChar) through Mark–Houwink (MH) constants. Detailed experimental conditions and data processing protocols are described elsewhere [16].

### 2.2. GPC Branching Index (gpcBr, Herein Abbreviated as Branching Index, B)

In principle, modern MD-GPC with highly sensitive detectors is capable of quantifying SCB (expressed as CH_3_/1000 C) by IR6 composition channels and thus LCB across MWD, provided a linear reference with a well-defined SCB type and LCB topology are available [9]. Unfortunately, these prerequisites for a direct analytical calculation of LCB frequency are unfulfilled due to backbiting during the high pressure polymerization process; thus, LDPE has a variety of types and amounts of SCB and LCB topologies. Branching index (*B*) was developed as a robust alternative. gpcBr, first introduced by Yau [19], enables quantitative assessment of total branching density (SCB + LCB) defined as

(1) $$\mathrm{gpcBr}={\left(\frac{\mathrm{Mw}\left(\mathrm{LS}\right)}{\mathrm{Mw}}\right)}^{a}\frac{\eta_{b}}{\eta_{L}}$$

where Mw(LS) and Mw are the bulk molecular weight from the LS detector and the backbone weight average molecular weight, respectively; [η]_b_ is the bulk intrinsic viscosity; and [η]_L_ is the intrinsic viscosity of a linear polyethylene homopolymer calculated from Mark–Houwink relationship ([η]_L_ = K·Mw^MHa^). To avoid possible notational conflict with the parameter α used in subsequent expressions, the Mark–Houwink exponent is denoted MHa throughout this work.

In addition, GPC related parameters were computed from MWD using the data-processing software GPCOne^TM^ (Trademark of PolymerChar, version 3.4) as Mz (z average molecular weight), bulk intrinsic viscosity (IV), Mp (molecular weight at the MWD peak), and Mv (viscosity-average molecular weight).

### 2.3. Melt Strength Measurement

Melt strength (MS) was measured using a Göttfert Rheotens (GÖTTFERT Werkstoff-Prüfmaschinen GmbH, Buchen, Germany) 71.97 coupled to a φ16 twin-screw extruder. Pellets were loaded into the capillary barrel and equilibrated at 190 °C for 10 min. The melt was extruded vertically through a capillary die with 30 mm length and 2 mm inner diameter (L/D = 15:2). The extrudate was drawn by counter-rotating serrated rollers with a 0.4 mm gap, which were positioned 100 mm below the die exit. Testing commenced at an initial draw velocity of 20 mm/min with a constant acceleration of 12 mm/s^2^. Force was recorded as a function of wheel speed using Rheotens Evaluation 2007 macros. Five replicate measurements were measured for each sample.

### 2.4. Metric Derivation of Melt Strength

Melt strength is conventionally defined as the maximum force sustained by an extruded polymer strand under a constant extrusion rate and linearly increasing draw speed prior to rupture [1,5]. However, no universally accepted protocol governs its measurement or data processing. Force–speed curves are inherently noisy owing to non-uniform melt flow, melt fracture, and oscillatory draw resonance, while temperature fluctuations, strand-diameter variations, and uneven cooling introduce additional variability. Although replicate measurements (typically five) are performed to improve reliability, the absence of standardized criteria for data extraction and averaging perpetuates inconsistency across studies, hindering both the establishment of unified analytical rules and the development of AI-based predictive models. To address this, we adopted a systematic feature-extraction approach, deriving six physically interpretable descriptors from the force–rotational-speed curves that collectively capture steady-state response, dynamic transitions, toughness, and failure characteristics during extensional flow.

### 2.5. Training and Validation Set Construction

The dataset consisted a total of 15 LDPE samples (75 sets of measurement data) produced by multiple manufacturers using a tubular reactor setup. Supplementary Materials Table S1 lists the characteristics of the LDPE samples. To ensure a robust and unbiased evaluation of model generalizability while accounting for potential inter-batch variability, we adopted a repeated random cross-sampling approach. Specifically, the full dataset was randomly partitioned into a training set (80%, *n* = 60) and a test set (20%, *n* = 15) in each trial. This procedure was independently repeated for 20 iterations. In every iteration, all the data preprocessing steps including feature scaling and any derived parameter calculation, were performed exclusively on the training subset to prevent overfitting. The same trained model was then evaluated on the held-out test subset. This strategy allows for a statistically meaningful assessment of model performance across diverse data partitions without relying on a single arbitrary split.

## 3. Results

### 3.1. Identification of the Critical Molecular Structural Parameters (Constraints) Using Entire MWD Spectrum for MS Model

To predict melt strength comprehensively, the proposed formulation integrates eight GPC-derived descriptors: Mw, Mn, PDI, Mz, IV, Mp, Mv and Branching Index(B) (Detailed definitions are available in the Supplementary Materials). Mw captures average chain length and, through its correlation with bulk intrinsic viscosity in the absence of LCB, reflects the entanglement density underpinning melt strength. PDI and Mz encode distribution breadth and the long-tail contribution, both of which govern the entanglement network resisting deformation under extensional flow. Collectively, these descriptors cover molecular weight, distribution breadth, and branching topology. As parameters conventionally adopted for characterizing polyolefin molecular features from both theoretical and empirical perspectives, they have potential to reflect melt strength-related properties.

**Figure 1:**
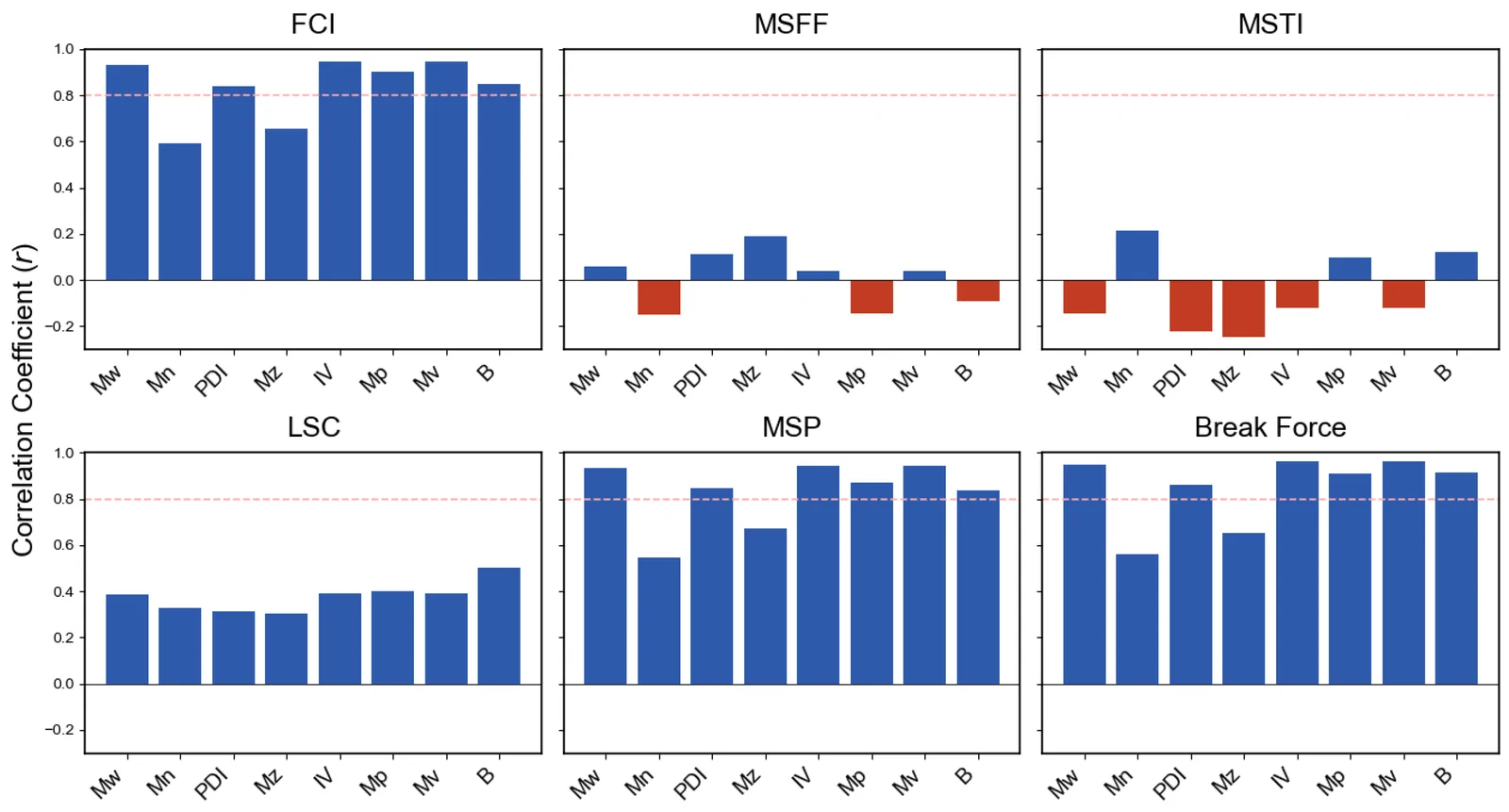
Correlation coefficients between the six melt strength features (defined in Table 1) and eight key parameters of the MWD. Blue lines denote positive correlations, and red lines denote negative correlations. The 0.8 significance threshold (|*r*|) is shown by dotted pink lines.

Figure 1 summarizes the Pearson correlation coefficients (r) between eight MWD derived parameters (Mw, Mn, PDI, Mz, IV, Mp, Mv and B) and the six extracted MS features (Table 1), with |*r*| ≥ 0.8 denoting a strong correlation. MSP, FCI, and Break Force (F_break_) correlate strongly with molecular weight descriptors (Mw, IV, Mv and Mp), distribution-breadth metrics (PDI and HMW), and branching index (B). These findings are consistent with the established roles of MWD breadth [20,21] and branching architecture [22] in governing polyethylene extensional rheology. Surprisingly, Mz—classically considered primary handle for enhancing MS by enriching the high molecular fraction—shows only modest correlations. This may stem from various possibilities, such as dataset limitations, or point to an intrinsic lack of correlation between Mz and MS amid the complex branching structures of LDPE. Conversely, MSFF, MSTI, and LSC exhibit no significant correlation with any MWD parameter (|*r*| < 0.5). By identifying the most predictive structural descriptors (|*r*| > 0.8), Figure 1 provides a physically motivated rationale for feature selection and guides the subsequent physics-informed machine learning strategy, in which fundamental rheological principles are systematically embedded within a data-driven framework.

Rather than modeling individual melt strength metrics (e.g., FCI, Fbreak, MSTI, MSP, MSFF, LSC)—which each capture only a partial aspect of melt extensional behavior, thus this paper utilizes a composite surrogate index as the target variable. Since no single observable can fully represent the overall melt strength performance of polyolefins, this surrogate index (MS) integrates the shared structural dependencies across multiple experimental measurements. This creates a single, unified target variable that offers a more holistic and robust foundation for structure–property modeling.

### 3.2. Identification of High-Molecular-Weight (HMW) Fraction

**Figure 2:**
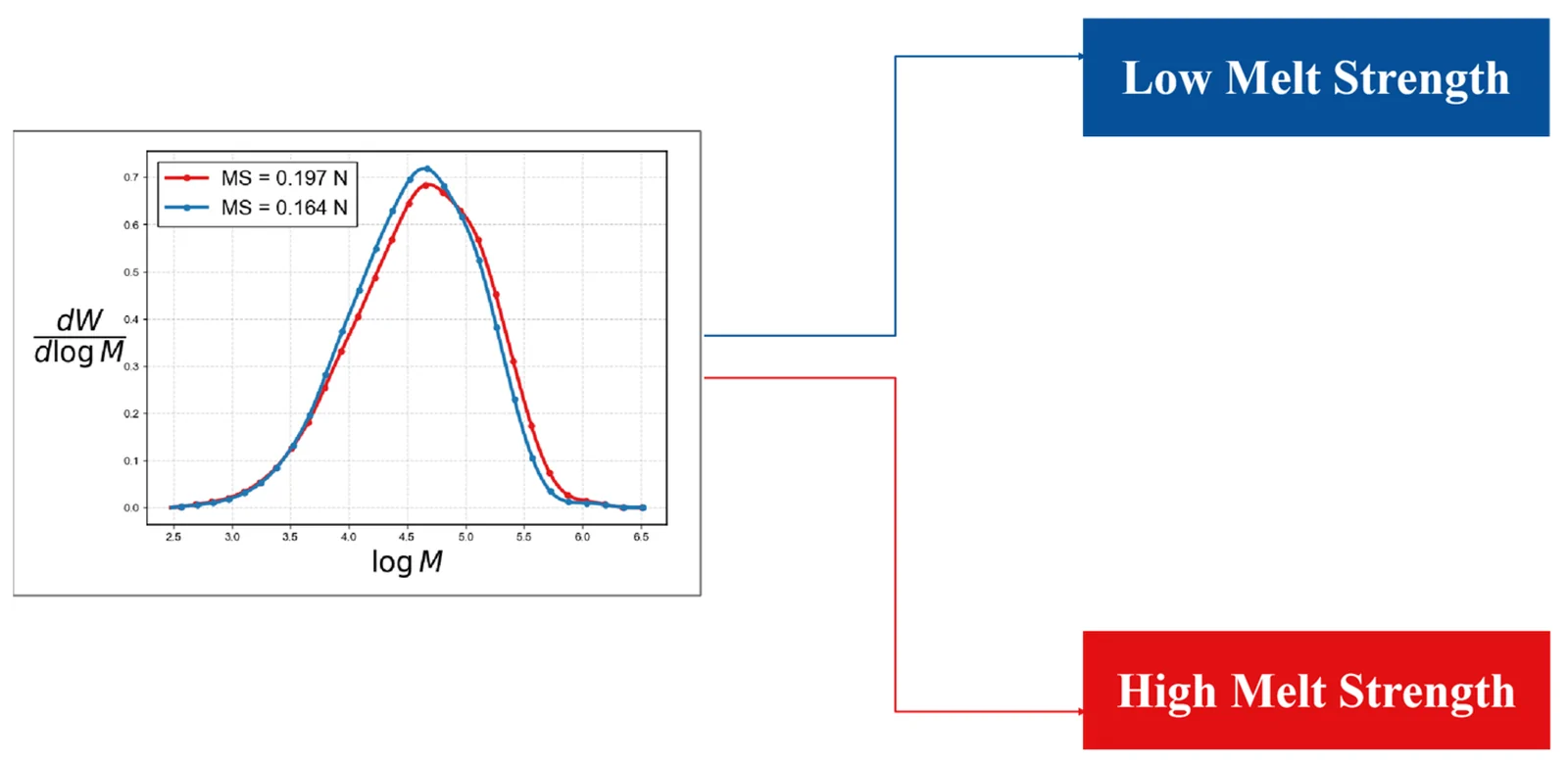
MWD examples of two LDPE samples faithfully capture structural differences that underlie distinct melt strengths.

Figure 2 displays the MWD of two LDPE samples exhibiting high and low MS, respectively. MS is correlated with MWD features; this effect arises mainly from the high-molecular-weight fraction, which increases the density of chain entanglements. Conversely, a broad, asymmetric MWD skewed toward low molecular weights dilutes the entanglement network and weakens MS. Thus, the critical determinant of MS is not a single average (Mw or PDI), but the detailed architectural features encoded in the full MWD. Conventional scalar descriptors condense information from the MWD and offer limited discriminating power [23], particularly when parameters are strongly correlated.

**Figure 3:**
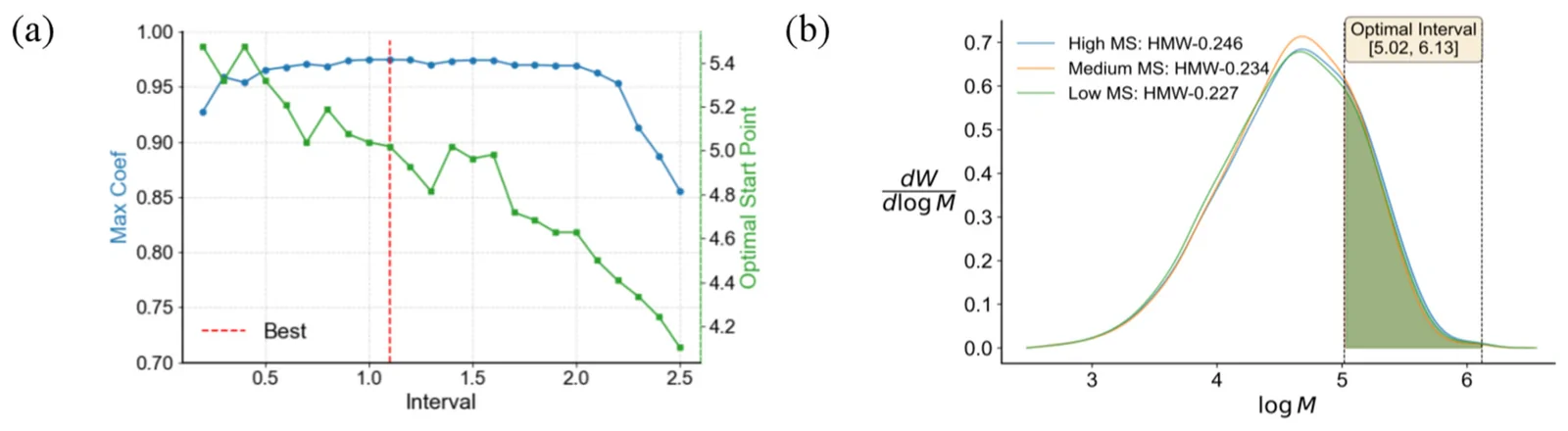
(**a**) Maximum correlation coefficient (the left Y-axis: Max Coef) and corresponding to the start point of optimal integration (the 2nd Y-axis) as a function of interval length of logM. (**b**) MWD curves for three LDPE samples with three levels of MS; shaded region indicates the logM interval [5.02, 6.13] yielding the strongest correlation between integrated MWD and MS.

To overcome this limitation, we bypassed predefined conventional descriptors and instead extracted physics-informed features directly from the full MWD profile. High-Molecular-Weight (HMW) index was defined as the trapezoidal integral of the MWD curve over the optimized interval [5.02, 6.13] in log M (backbone molecular weight) space, requiring no additional calibration or model assumptions. This interval yields a Pearson correlation (r) of 0.975 with MS (Figure 3 with the correlation coefficient (*r*) > 0.95 across the entire samples of data) and consistently resolves the HMW tail for samples spanning low, medium, and high melt strength, where subtle differences in long-chain content are most pronounced.

This HMW window corresponds to backbone molecular weights of approximately 10^5^–10^6^ g/mol, representing the fraction of chains that surely form effective entanglements under the extensional conditions of MS testing [5,11]. These long chains govern strain hardening and affect zero-shear viscosity, the principal mechanisms underlying melt strength and resistance to sagging during and after processing. Accordingly, HMW index is not merely an empirical fitting parameter; it quantitatively represents the proportion of molecular species that actively sustain the entanglement network and extensional response.

### 3.3. Identification of Descriptors via Physics and Data Guided Learning

To quantitatively relate molecular architecture to MS, we derive an analytical expression informed by the full MWD and entanglement theory:

(2) $$MS=f_{phys}\left(G;\omega \right)$$

where *G* and ω denote the MWD-derived feature vector and the trainable parameter respectively. The function fphys is structured according to physical mechanisms to preserve mechanistic interpretability.

Existing data-driven approaches, including black-box models and physics-informed neural networks (PINNs) [24,25], can improve generalization by embedding physical laws, especially when data sets are limited or noisy [26]. However, their neural network backbones often obscure how molecular descriptors determine macroscopic properties, limiting their usefulness for inverse design.

We therefore embed a physics-based analytical expression—a parameterized extension of the Mark–Houwink (MH) equation using intrinsic viscosity as a proxy for chain topology and MS—directly into the model output. This creates a fully differentiable pipeline from MD-GPC features to MS prediction. The model is trained end-to-end using automatic differentiation, allowing the physical expression to be optimized jointly with the learnable parameters. Because the physics-based module is active rather than merely a regularizer, the resulting model is more interpretable and better suited to inverse molecular design.

To distill the multidimensional structural information of LDPE into a single, physically meaningful metric, we developed a physics-guided data fusion approach that transforms six-dimensional structural parameters into a unified melt strength index. Specifically, we first screened MD-GPC-derived parameters to identify features exhibiting strong linear correlations with MS characteristics (|r| > 0.8). These included molecular weight descriptors (Mw, Mn), molecular weight distribution metrics (PDI, HMW), and chain branching indices (B) (Figure 1 and Figure 3). The selected features were subsequently normalized via min–max scaling to eliminate dimensional discrepancies. Feature weights were then assigned in proportion to their respective maximum correlation coefficients with MS and normalized to sum to unity. The final melt strength index was computed as the weighted sum of these normalized features, yielding a unified quantitative metric that encapsulates the essential structural determinants of melt strength for subsequent structure–property modeling.

(3) $$MS=\sum_{i}\omega_{i}\cdot {x_{i}}^{norm}$$

where ${x_{i}}^{norm}$ denotes the min–max normalized value of the i-th pre-selected MD-GPC descriptor (with |r| > 0.8), and $\omega_{i}$ denotes normalized feature weight, which is proportional to the maximum correlation magnitude of each structural descriptor, subject to $\sum_{i}\omega_{i}=1$. Hereafter, the term MS denotes this calculated melt strength index.

Various candidate models are constructed using five MD-GPC-derived features (Mw, PDI, B, HMW and Mz) previously identified as key physically meaningful descriptors of chain entanglement and topological constraints. Diagnostic scatter plots (details in Figure S1, Supplementary Materials) showed that MS exhibits distinct functional dependencies on these molecular descriptors:

1. MS shows a very strong linear correlation with Mw (r = 0.947, Figure 1). The logarithmic form is slightly weaker (r = 0.904, Figure S1, Supplementary Materials), suggesting a near-linear or higher-order power-law relationship. Power-law relationship is often used in polymer science for structure/property relationship [27,28]. Linear Mw terms are therefore prioritized, with logarithmic or exponential corrections included where appropriate.
2. Comparably strong correlations with HMW in both linear (r = 0.971) and exponential (r = 0.974) forms. Both forms are retained to allow for alternative physical interpretations.
3. A similar high correlation with B across all three forms (r = 0.90) without clear preference. Multiple functional representations are therefore included.

Only a weak correlation with Mz (r = 0.6). Mz is retained in selected models because of its physical relevance to chain entanglements.

These observations have justified restricting the model space to power-law, log-linear, and additive or multiplicative combinations that are consistent with both the observed data trends (Figure S1, Supplementary Materials) and physical interpretability. As shown in Table 2, starting from the simplest power-law baseline (Formula1: MS∝Mw^β^), we progressively incorporate the corrections for PDI, B and HMW. Formulas2 and 8 introduce exponential or power-law terms involving Mz/Mw to capture the effects of high-molecular-weight tail and B. Formulas3 and 9 add the correction terms for PDI and HMW, respectively. Formulas4, 6, and 11 construct linear superposition models in logarithmic space, whereas Formulas5, 7, 10, and 12 explore nonlinear responses by placing variables inside or outside exponential functions.

Moving beyond the limitations of standalone statistical metrics like RMSE and R^2^, we propose a composite scoring system integrating statistical accuracy, model complexity, and physical consistency to objectively identify optimal descriptor from extensive candidate library. First, the Akaike Information Criterion (AIC) and Bayesian Information Criterion (BIC) [29] are employed to balance goodness-of-fit with parameter count. Under the assumption of independent and identically distributed Gaussian of residual errors, maximizing the Gaussian likelihood is equivalent to minimizing the residual sum of squares (RSS). The Maximum Likelihood Estimate (MLE) of residual variance is ${\hat{\sigma }}^{2}=RSS/n$:

(4) $$AIC=n\cdot ln\left(RSS/n\right)+2k$$

(5) $$BIC=n\cdot ln\left(RSS/n\right)+k\cdot ln\left(n\right)$$

where n, k and RSS are sample size, the number of parameters, and the residual sum of squares respectively. Lower values indicate better models. To quantify physical consistency (S_phy_), we impose three validation criteria: monotonicity (MS should increase with Mw), limit behavior (the model should regress to linear chain behavior as PDI → 1.0), and physically reasonable parameter signs (e.g., entanglement indexes should be positive). A score of S_phy_ = 1.0 is assigned if all conditions are satisfied and 0.0 otherwise. The final Comprehensive Score is defined as the product of the normalized test set coefficient of determination (test R^2^) and the physical consistency score, weighted by the AIC likelihood:

(6) $$SCORE=\frac{1}{4}\left(R_{norm}^{2}+\left(1-RMSE_{norm}\right)+\left(1-AIC_{norm}\right)+S_{phy}\right)$$

where $X_{norm}=\frac{X-X_{min}}{X_{max}-X_{min}}$. Further model refining was conducted through systematic evaluation of candidate formulas using cross-validation and predictive performance metrics. We assessed whether the inclusion of each descriptor—and its specific functional form—significantly improved model fit, and iteratively adjusted the mathematical relationships based on empirical performance. After multiple rounds of optimization, the final scaling law was selected based on the balance of statistical significance, physical interpretability, and predictive accuracy, as summarized in Table 3.

The best performing models are Formula11 (SCORE = 1.0000), followed by Formula2 (0.9963), Formula8 (0.9930) and Formula12 (0.9924). These results reveal two effective routes to accurate MS prediction. First, Formulas11 and 9 show that explicitly capturing high-molecular-weight (HMW) fraction is highly effective: Formula11 reaches the Test R^2^ of 0.860 using HMW, while Formula9 at 0.855 with this single descriptor, making it especially suitable for laboratories using only conventional HT-GPC. Second, Formulas8 and 2 demonstrate that, despite the modest linear correlation between Mz and MS (r = 0.65, Figure 1), Mz can still improve prediction when combined with other descriptors through exponential or higher-order terms. It is worth noting that all the leading models satisfied the required physical constraints, ensuring both interpretability and reliable extrapolation.

Formula10 (SCORE = 0.9747) illustrates a case where added complexity is not justified. Although the Test R^2^ at 0.854 is comparable to several top models, its SCORE is lower than those of mid-tier models such as Formulas3, 4, and 1. This indicates that the complexity penalty outweighs its marginal gain in accuracy. Formula10 may therefore be useful in specialized cases, but its limited complexity/performance balance reduces its general applicability.

Formula1 (SCORE = 0.9753) ranks relatively high mainly because of its simplicity. However, its Test R^2^ of 0.682 using Mw is much lower than that of Formula9 (0.855), reinforcing that the HMW descriptor captures the entanglement density more effectively than weight average molecular weight. This comparison shows that physically relevant descriptors are more important than structural simplicity alone.

The mid-tier models, including Formulas6, 3, and 4 (SCORE between 0.9733 and 0.9759), maintain reasonable generalization but are limited by their reliance on conventional descriptors. They capture basic physical trends but lack the accuracy gained by targeting the high-molecular-weight region or using optimized higher-order terms.

**Figure 4:**
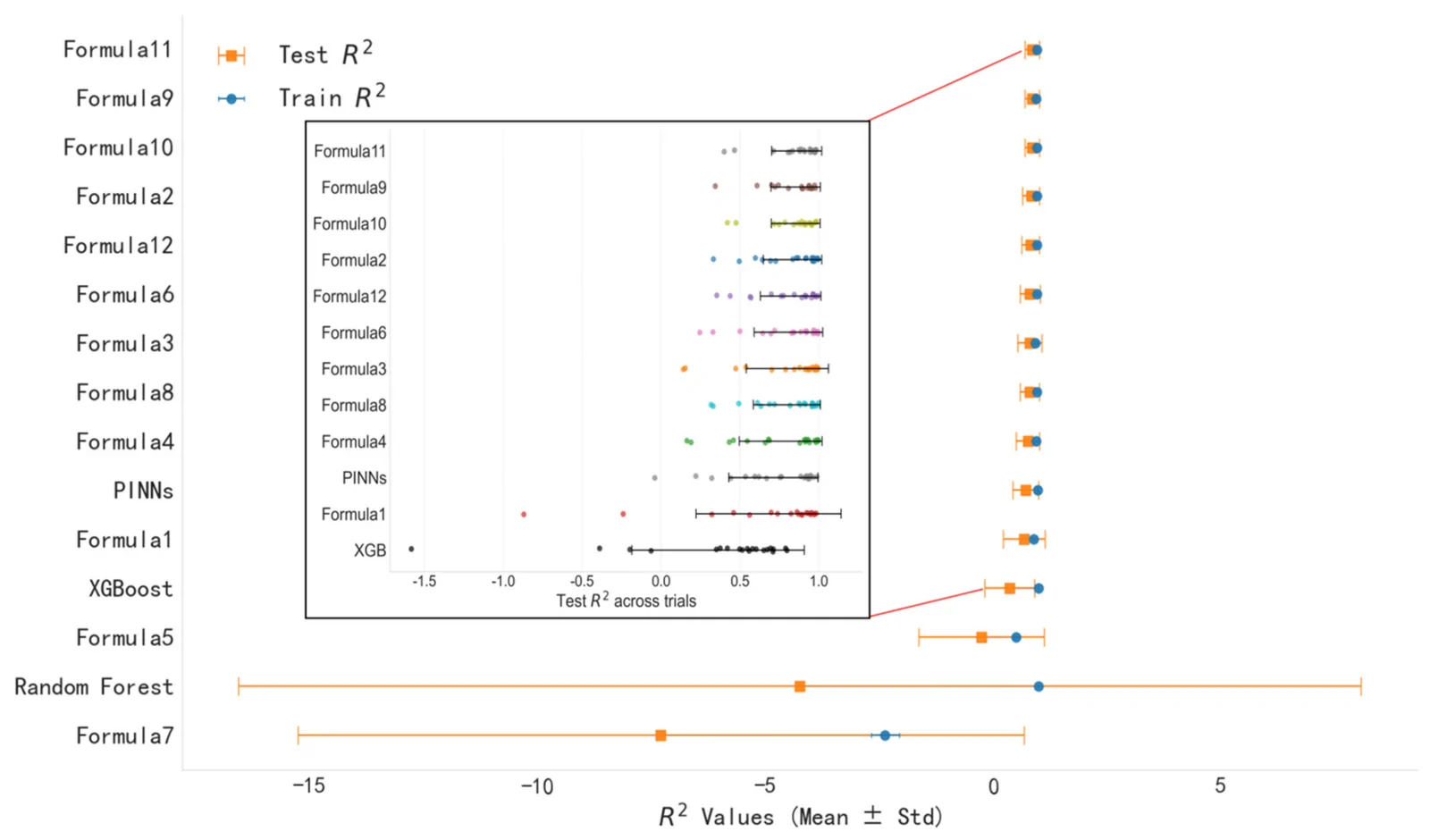
Distributions of test-set R^2^ (Test R^2^) and training-set R^2^ (Train R^2^) values for MS prediction models over 20 train–test splits. Insets show magnified the views of the top-performing models based on test-set R^2^. In insets, black bars denote mean±standard deviation, and colored dots represent individual split results.

We benchmark the formulas against PINNs, XGBoost (also abbreviated as XGB in Figure 4), and Random Forest to further evaluate their predictive performance. Figure 4 highlights the poor generalization of unconstrained black-box models. Although PINNs (SCORE = 0.7468), XGBoost (SCORE = 0.4366), and Random Forest (SCORE = 0.3752) fit the training data nearly perfectly (R^2^ > 0.97), their test-set performance collapses, with Random Forest reaching R^2^ = −1.947. In the absence of mechanistic constraints, these models tend to capture spurious correlations rather than true molecular property relationships, demonstrating structural plausibility being more important than raw training accuracy under realistic conditions.

For example, Formula7 (SCORE = 0.3978) ranks among the worst-performing models and provides a clear warning: blindly introducing higher-order moments, such as treating Mz as dominant without proper transformation, amplifies noise rather than signal, especially given the weak linear correlation between Mz and MS. Its test R^2^ of −7.291 and test MAPE of 10.62% show that including poorly justified descriptors can lead to severe model failure.

Among competing candidate models with comparable predictive scores in Table 3, Formula11 (SCORE = 1.0000, Test-set R^2^ = 0.860) is ranked as the optimal model. It achieves a favorable balance of physical interpretability and model parsimony. This equation is an extension of the base Mark–Houwink (MH) relationship. The HMW descriptor is preferred over Mw due to its greater physical relevance, and the model enables effective decoupling of the intertwined effects of HMW and PDI. The resulting correlations align better with the underlying physical mechanism and provide a more plausible description of the experimental observations. Subsequent analysis and inverse design will be performed based on this model.

It is noted that when constructing prediction models over 20 independent train-test splits, we enforced grouping constraints to ensure that all replicates belonging to the same parent sample were kept entirely within either the training set or the test set. No replicates from a single sample were split across the two subsets, thus avoiding artificially inflated predictive performance caused by leakage between replicates.

To contextualize the performance of these composite MS models, we also evaluated predictive models trained directly on the individual raw metrics: FCI and Break Force. Both models were constructed using the identical pipeline described in the main text. Despite strong linear correlations between FCI/Break Force and GPC features, the predictive performance of these individual models was inferior to that of the composite MS model. Notably, while the Break Force model achieved acceptable training performance, its test-set performance was sub-optimal, indicating limited generalization capability. Furthermore, the substantial variance in both training and test metrics across the 20 splits suggested that predictions based solely on FCI and Break Force lack robust reliability. Detailed results, including the mean and standard deviation of training and test R^2^ and RMSE across 20 repeated group-aware splits, are provided in the Section “Performance of Predictive Modeling with Break Force and FCI” of the Supplementary Materials.

## 4. Discussion

### 4.1. Sensitivity and Interpretability Analysis

**Figure 5:**
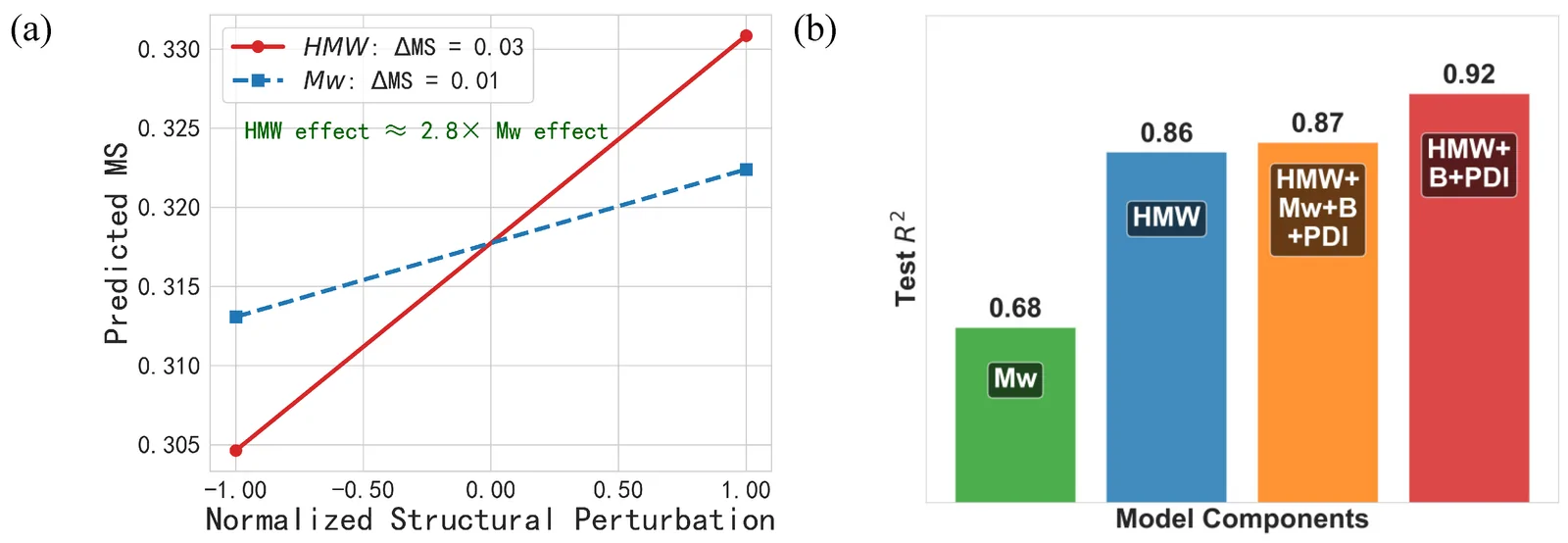
(**a**) Local sensitivity analysis with linear regression shows HMW has a much stronger influence on MS than conventional Mw. (**b**) Ablation study: HMW replaces Mw (R^2^ at 0.68 → 0.86); adding B and PDI boosts R^2^ to 0.92; reintroducing that Mw lowers it to 0.87, confirming HMW as the key descriptor.

Figure 5 presents a systematic comparison of the evaluated candidate models under identical training and testing conditions. Across all metrics, models employing HMW as a molecular descriptor substantially outperform their counterparts based on conventional Mw. Notably, replacing Mw with HMW increases the test-set R^2^ from 0.68 to 0.86, representing a marked improvement in predictive fidelity and underscoring the superior capacity of HMW to capture the underlying structure–property relationships governing the target response. This agrees with industrial polyolefin practice, where high MS is promoted by high-molecular-weight components, especially in the absence of LCB [30]. In models already containing B and PDI, the concurrent use of HMW and Mw reduces, rather than enhances, the model’s predictive performance, indicating that Mw is redundant once HMW is included and may introduce noise.

**Figure 6:**
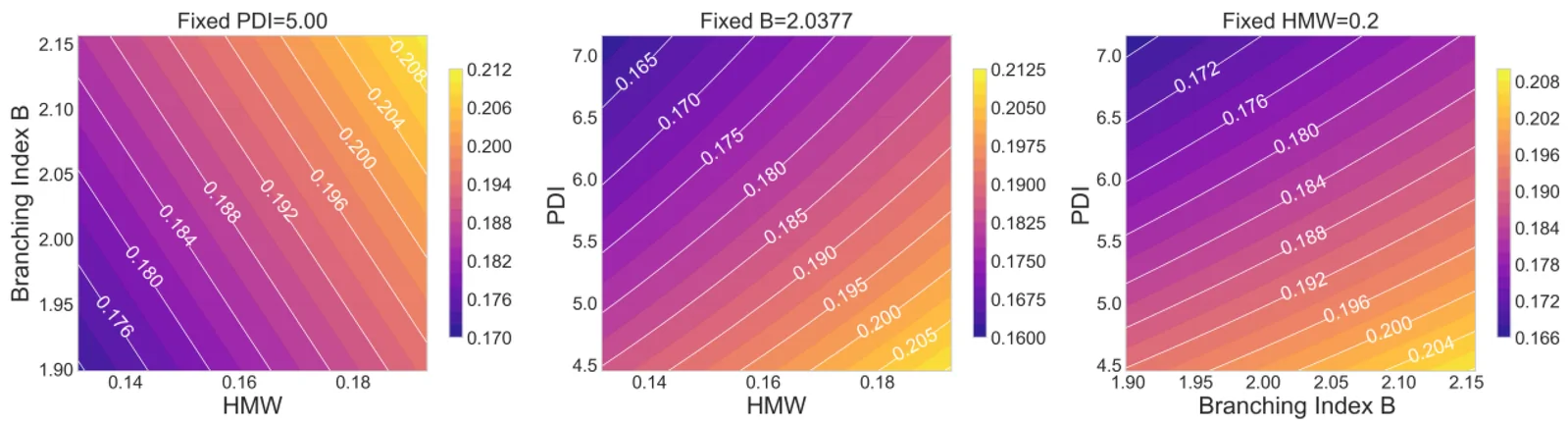
Hierarchical Sensitivity Analysis of MS via Formula11 model: HMW as the primary driver, PDI as a secondary feature, and limited role of B (Branching Index).

Figure 6 shows a high-dimensional sensitivity analysis based on the interpretable Formula11 model. HMW is the dominant determinant of MS across the parameter space. At fixed PDI and Br, MS increases monotonically and nonlinearly with HMW, indicating that high-molecular-weight chains are the main contributor to an effective entanglement network.

PDI has a secondary but significant effect. At same HMW, MS decreases as PDI increases, reflecting the dilution of entanglements by low-molecular-weight species. This effect is partly weakened at high level of HMW but not eliminated, indicating that high level of HMW can only partially offset the negative influence of broad molecular weight distribution.

Branching index (B) has the weakest influence on MS. Only a marginal positive trend appears at very high B, and the response surface is relatively flat along the B direction. For the tubular reactor LDPE studied here, B consists of both short-chain and long-chain branching, therefore contributes only modestly to MS. Excessive long-chain branching (such as highly branched chains) may thus introduce processing or homogeneity penalties with limited performance benefit.

Overall, the unified descriptor provides both accurate prediction and clear interpretation. It reveals a hierarchical structure–property relationship: HMW is the primary driver of MS, PDI is secondary, and B has a comparatively small effect. For high MS LDPE design, strategies should prioritize increasing HMW and limiting PDI, while overly tight control of branching (B) may offer limited additional benefit.

### 4.2. Inverse Design with Physics-Guided Conditional Diffusion Model

In inverse design, given a target MS ($y_{target}$), we seek the parameters of molecular structures that achieve this target through the trained physics-guided forward model. Diffusion models are well suited to this task because they learn the distribution of feasible designs rather than performing point-wise optimization. Our framework couples this generative model with the analytical forward model, guiding sampling toward physically meaningful regions of molecular parameter space. To suppress implausible outputs from stochastic generation, we apply a conditional filter based on p(PDI|HMW), which confines PDI within physically admissible lower and upper bounds conditioned on high-molecular-weight tail magnitude, constraining generated chain topology to follow established polymer physics correlations.

We therefore propose a physics-guided conditional diffusion model for inverse design of LDPE MS. For $y_{target}$, to avoid local minima (common in gradient-based optimization), it directly generates molecular topology vectors: $z=G^{T}\in {\mathbb{R}}^{n}$, where n denotes the dimensionality of the key descriptors. The forward process adds Gaussian noise over T steps:

(7) $$z_{t}={\sqrt{\bar{\alpha }}}_{t}z_{0}+\sqrt{1-{\bar{\alpha }}_{t}}\epsilon ,\;\epsilon \sim N\left(0,\;I\right)$$

where ${\bar{\alpha }}_{t}=\prod_{s=1}^{t}\alpha_{s}$ governs the relative contribution of the original informative signal versus injected random noise throughout the generation process. The reverse process is parameterized by a neural network $\epsilon_{\theta }\left(z_{t},t,y\right)$ that predicts the added noise [31].

The model also incorporates the validated forward relationship $\hat{y}=f_{phys}\left(z_{0}\right)$ to map structure descriptors to MS, yielding a physics-consistency loss term. The training objective is

(8) $$L_{total}=E_{z_{0},y,t,\epsilon }||\epsilon -\epsilon_{\theta }\left(z_{t},t,y\right)|{|}^{2}+\lambda_{phys}\cdot E_{z_{0},y}[(\hat{y}-y{)}^{2}]$$

where the 1st term is the standard diffusion loss, the second term enforces physical consistency via $\hat{y}=f_{phys}\left(z_{0}\right)$, with λ balancing the two terms [32,33,34,35]. During inference, classifier guidance aligns candidate with the target MS. If *ŷ* is the predicted MS at an intermediate noisy state, the guided prediction is

(9) $${\tilde{\epsilon }}_{\theta }=\epsilon_{\theta }\left(z_{t},t,y_{target}\right)-s\cdot \nabla_{z_{t}}{\left({\hat{y}}_{t}-y_{target}\right)}^{2}$$

where s≥0 is the guidance scale. This encourages candidate to satisfy the target MS while preserving diversity.

Importantly, all generated samples are subject to feasibility filtering: using the GPC-trained conditional model *p*(PDI∣HMW)—a regularized shallow neural network outputting *μ*(HMW) and log*σ*^2^(HMW)—we enforced hard constraints such that only samples with PDI ∈ [*μ* − *3σ*, *μ* + *3σ*] (covering > 99.7% of observed industrial combinations) and branching index B within empirically calibrated GPC–viscometry bounds are retained [36] (Figure S2, Supplementary Materials).

### 4.3. Inverse Design Capability for Target Melt Strength

**Figure 7:**
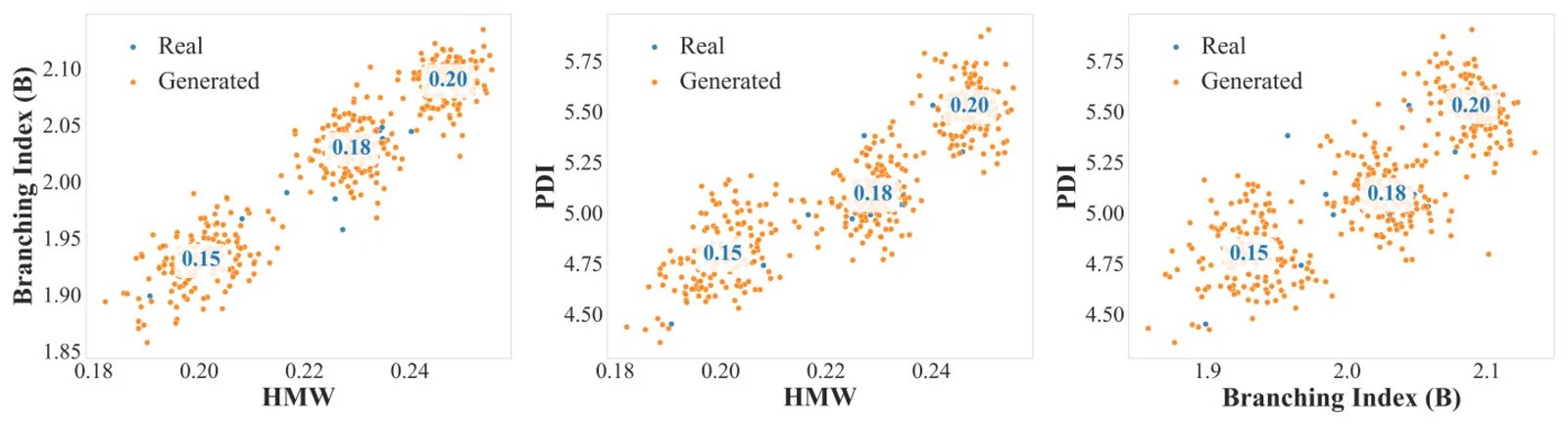
Joint distributions of real experimental data (blue) and diffusion-generated samples (orange) across three key descriptors for target MS values of 0.15, 0.18, and 0.2 N respectively.

The proposed diffusion probabilistic model learns the underlying distribution of the high-dimensional material parameter space from the training dataset. It captures the nonlinear couplings among HMW, B and PDI, generating physically consistent and diverse candidate structures in an unsupervised manner (Figure 7). As MS increases, HMW and B exhibit a synergistic growth trend overall. However, in the 2D projections involving PDI, while the overall mean rises with MS, there is significant overlap among the clusters corresponding to different MS values along the PDI dimension. This phenomenon reveals the complexity of the material’s microstructure: although high molecular weight or high branching level is typically associated with a broader molecular weight distribution (i.e., higher PDI), the system tends to suppress polydispersity by optimizing topological structures when pursuing higher MS (i.e., for given HMW and B, a higher MS often corresponds to a lower PDI).

**Figure 8:**
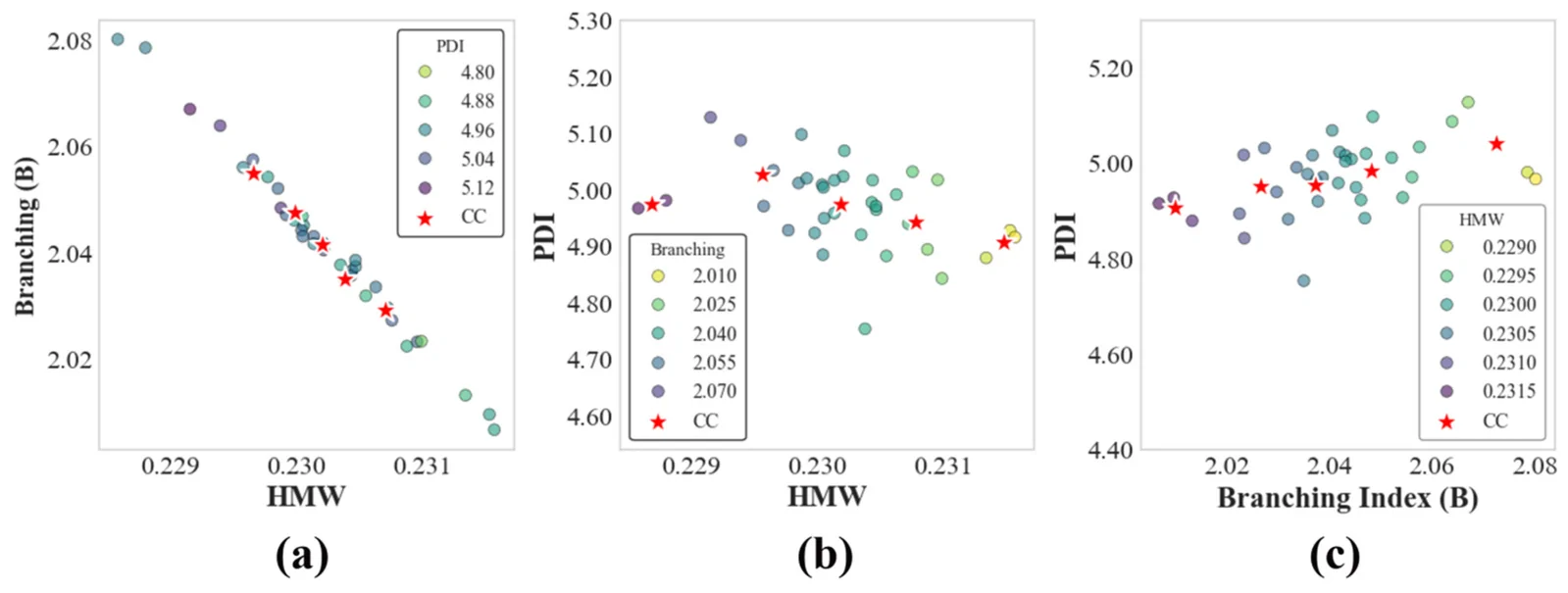
Projections of diffusion-generated results in the HMW–Branching (B)–PDI space. Colors represent the third variable’s magnitude. Red stars indicate the Cluster Centers (CCs) for the five magnitude-based groups (High to Low). (**a**) HMW-Branching(B) projection; (**b**) HMW-PDI projection; (**c**) Branching(B)-PDI projection.

When the target MS is set to 0.18 N, the diffusion-based model generates a diverse spectrum of viable structure combinations that meet the required performance threshold, in contrast to conventional approaches that typically return a single point estimate (Figure 8). These diverse design pathways stem from the compensatory effects among polymer chain parameters. As shown in Figure 8a, examining the evolution trajectory of the cluster centers (CCs) reveals that for the same target MS, a higher HMW lowers the requirement for the branching index (B). This indicates that sufficient high-molecular-weight components are adequate to build an effective molecular chain entanglement network, thereby reducing the reliance on the degree of long-chain branching while still achieving the target MS.

Figure 8b reveals that the interplay between HMW and PDI exhibits complex, non-monotonic fluctuations. This observation likely reflects the constrained variation in HMW at the target MS, yet it highlights a critical practical insight: simultaneously matching HMW and PDI demands condition dependent trade-offs that must be balanced within the feasible processing window.

**Figure 9:**
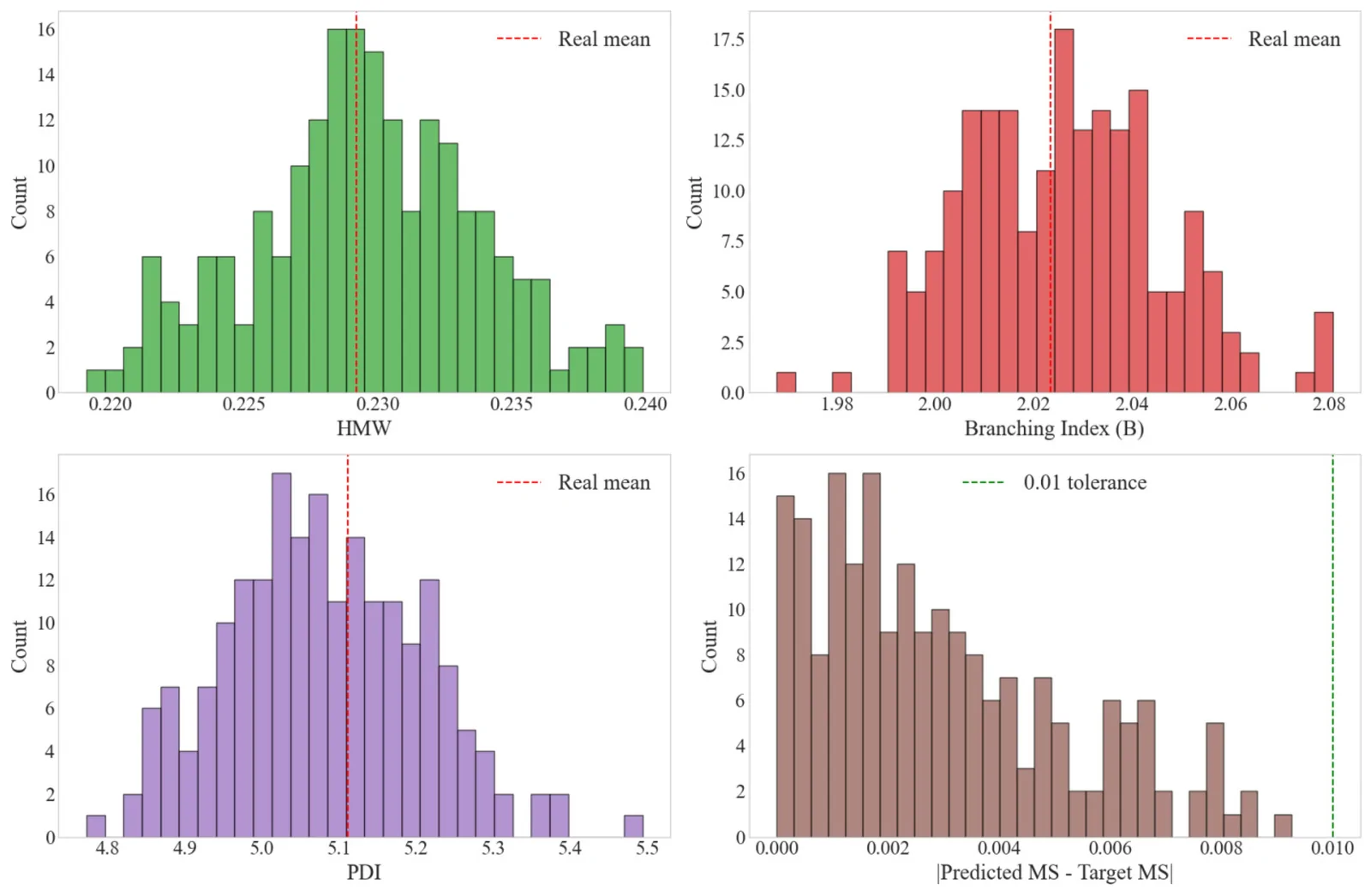
Multi-modality in single-target (MS = 0.18) inverse design. 200 feasible design candidates are generated with histograms of their parameters and predicted MS errors.

Notably, every candidate generated by the diffusion-based inverse design model satisfies the predefined physical constraints, with predicted MS errors tightly clustered within ±0.01. (Figure 9). The diversity of viable parameter combinations achieved under such stringent performance criteria provides considerable flexibility for downstream engineering decisions. Practitioners can further refine or screen these candidates in light of real-world factors—including polymerization processing constraints, cost considerations for resin selection and blending, and manufacturing operation windows—thereby tailoring LDPE formulations to specific application requirements with high precision.

### 4.4. Generalizability and Limitations

To systematically assess the robustness of the proposed framework under broader application scenarios, we applied the model—without any modification to its parameters—to experimental datasets collected from multiple commercial sources of LDPE 2426H grade produced by different manufacturers in China (Table S2, Supplementary Materials), as well as samples processed under varying industrial conditions. On these additional test sets, the model achieved a coefficient of determination (R^2^) of 0.6423, a root-mean-square error (RMSE) of 0.0077, and a mean absolute percentage error (MAPE) of 2.76%. These results demonstrate that the framework not only exhibits transferability across LDPE grades within the tubular process but also maintains strong adaptability to real-world variations in feedstock and processing conditions, underscoring both the physical generality and the engineering relevance of the underlying modeling paradigm.

To further evaluate the model’s generalizability across distinct polymer systems, we transferred the formulation originally developed on the LDPE grade 2426H dataset directly to another commercial LDPE grade 1810D (also made in tubular process). Remarkably, without any parameter adjustment, the original model exhibited reasonable predictive accuracy on 1810D, indicating a baseline level of transferability. More impressively, only minor recalibrations of two physically interpretable parameters—those governing the baseline offset and the branching contribution—were sufficient to achieve excellent predictive performance on the new system. This minimal recalibration reduced the mean absolute error (MAE) from 0.035 to 0.011 and the root-mean-square error (RMSE) from 0.039 to 0.013, while substantially improving the coefficient of determination. The successful transfer hinges on adjustments to only two parameters: the intercept α and the branching-related coefficient δ. Specifically, α required a modest 12% reduction from its original value, while δ was refined to better capture LDPE 1810D-specific topological effects. This limited recalibration demonstrates that the core functional form of the model is robust across LDPE grades, with systematic shifts largely absorbed by physically meaningful degrees of freedom (data and model transfer procedures are detailed in Supplementary Materials).

While the present study demonstrates the efficacy of the proposed diffusion-based inverse design framework for LDPE molecular architecture optimization, several limitations warrant acknowledgment and outline directions for future investigation. First, the current model is trained and validated primarily on tubular process LDPE systems within a defined domain of molecular weights (Mw < 2 × 10^6^ g/mol) and polydispersity indices (PDI ≤ 7); extrapolation to fundamentally different polymer chemistry (e.g., polypropylene or metallocene-catalyzed polyethylene) or to autoclave process products with distinct branching topologies will require dedicated retraining and experimental verification. Second, although the forward model exhibits strong cross-grade transferability and the inverse framework shares the same physically grounded descriptors, closed-loop experimental validation of the generated candidate structures remains to be performed in subsequent work. Third, the present framework relies on a finite experimental dataset; expanding the training corpus to encompass a broader range of industrial grades, processing conditions, and end-use performance metrics will further enhance the model’s generalizability and reduce uncertainty in underrepresented regions of the design space. Finally, integrating multi-objective optimization—simultaneously balancing rheological performance, processability, mechanical properties, and economic feasibility—represents a natural extension that will bring the framework closer to fully autonomous, industry-ready polymer design. Addressing these challenges in future work will progressively expand the domain of applicability and accelerate the translation of data-driven molecular design into scalable manufacturing practice.

## 5. Conclusions

We report a unified molecular descriptor that quantitatively links the architectural features of LDPE including high-molecular-weight (HMW) fraction, branching index (B), and polydispersity index (PDI)—to its melt strength (MS). This descriptor emerges from a physics-guided machine learning framework that synergistically integrates domain-specific rheological knowledge with a diffusion probabilistic model.

To the best of our knowledge, this work represents the first systematic, data-driven modeling study dedicated to unraveling the quantitative structure–MS relationships in LDPE across a diverse molecular design space.

By embedding physical priors into the learning process, the physics-guided framework not only enforces known constraints but also reveals compensatory mechanisms among molecular parameters—most notably, the dominant and often decisive role of the high-molecular-weight fraction (HMW). Our analysis qualitatively identifies HMW as the primary architectural driver of melt strength, outweighing the contributions of branching or polydispersity among the LDPE features investigated in this study. This insight aligns with entanglement theory that remains elusive in quantitative form due to the coupled nature of polymerization kinetics.

Thereby, the framework provides interpretable design pathways that are often inaccessible to purely data-driven deep learning models. Leveraging this structure, the diffusion model serves as an efficient sampler of the multi-modal inverse design space, enabling the generation of diverse, physically feasible formulations for target MS values without requiring iterative experimentation. The success of this approach underscores the critical value of cross-disciplinary integration: combining mechanistic insight with generative AI yields models that are not only accurate but also transparent and actionable.

It should be noted that independently tuning HMW, PDI, or branching while holding other molecular features fixed remains experimentally challenging in real polymerization processes—making comprehensive closed-loop validation difficult at scale. Nevertheless, the model’s key insight—that the high-molecular-weight tail (HMW) dominates melt strength far more than PDI or branching—offers clear directional guidance for industrial formulation: when resources are constrained, prioritizing HMW enhancement yields greater performance gains than fine-tuning long-chain branching.

Given these practical constraints, our current validation is necessarily simulation-based. Future work must therefore couple this framework with pilot-scale or industrial experiments to assess real-world processability, stability, and performance as feasible targeted trials become available.

Although theoretical simulations—such as CFD modeling [37]—offer abundant data, they often lack the operational noise, batch variability, and processing context inherent in real production. Nevertheless, as digitalization accelerates data accumulation in manufacturing, we anticipate an emergent regime in which sufficient data volume and diversity may gradually reduce reliance on explicit physical priors. In such a regime, physics-guided diffusion models could evolve toward truly unified, self-adapting design engines tailored to industrial practice.

## 6. Patents

A Chinese patent application (Application No. CN 202610784196.0, title: A Method and Apparatus for Evaluating Melt Strength of Polyolefin) based on the research reported in this article is pending.

## Figures and Tables

**Table 1:** Definitions of six physically interpretable melt strength features extracted from the force–rotational-speed curves of Gőttfert Rheotens MS measurement.

Metric	Physical Interpretation	Mathematical Formula
Flat Curve Index (FCI)	Steady-state MS under quasi-equilibrium extension	$\mathrm{FCI}\;=\;\frac{1}{N_{P}}\sum_{i\in P}{F(v}_{i})$ P: Set corresponding to the plateau region of the smoothed force–speed curve. $N_{P}$: Number of data points in set P ${F(v}_{i})$: Measured tensile force at speed $v_{i}$
Melt Strength Feature Frequency (MSFF)	Dominant frequency extracted from continuous wavelet transform	$f_{\mathrm{MSFF}}\;=\;\frac{f_{0}}{a_{\mathrm{peak}}}$ $f_{0}$: Mean relaxation frequency derived from the wavelet transform $a_{\mathrm{peak}}$: Dominant relaxation frequency from the wavelet transform
Melt Strength Toughness Index (MSTI)	Ratio of the area under the force–speed curve to the peak force	$\mathrm{MSTI}\;=\;\frac{\int_{o}^{v_{\max}}F(v)\mathrm{dv}}{F_{\mathrm{break}}}$ $F_{\mathrm{break}}$: Peak force on the force–speed curve
Melt Strength Phase Transition Force (MSP)	Marks the transition from elastic- to plastic-dominated response	$F_{\mathrm{MSP}}{=F(v}_{\mathrm{MSP}})$ vMSP=min{v| F″=0 & dF″dv > 0}
Linear Segment Curvature (LSC)	Early-stage nonlinearity in the flow response	$\mathrm{LSC}=\frac{1}{N_{P}}\sum_{i\in P}\frac{{|F}^{\prime \prime }{(v}_{i})|}{{{[1+F^{\prime \prime }(v}_{i}{)}^{2}]}^{3/2}}$
Break Force (Fbreak)	Reflects the ultimate load-bearing capacity of the melt	$F_{\mathrm{break}}{=\max}_{v}F(v)$

**Table 2:** Candidate physics-guided models for MS prediction (α, β, γ, δ, λ, η, σ, δ and κ are all learnable parameters).

Model	Mathematical Expression	Physical Motivation
Formula1	${\mathrm{MS}=\alpha \mathrm{Mw}}^{\beta }$	Base Mark-Houwink (MH).
Formula2	$\mathrm{MS}=\frac{{\alpha \mathrm{Mw}}^{\beta }}{{\mathrm{PDI}}^{\gamma }}\;\times \;(1+\eta B)\;\times \;\exp(\delta \cdot \left(\mathrm{Mz}/\mathrm{Mw})\right)$	Adds corrections for: branching, polydispersity and high MW tail via Mz/Mw.
Formula3	${\mathrm{MS}=\alpha \mathrm{Mw}}^{\beta }{\;\times \;\mathrm{PDI}}^{\gamma }$	Extends MH by coupling Mw and PDI multiplicatively.
Formula4	MS=exp(α+βln(Mw) − γln(PDI)+ δ·clip(Mz/Mw))	Log-linear with Mz/Mw and imposes linear penalty.
Formula5	$\mathrm{MS}=\alpha \cdot \exp(\beta \mathrm{Mw}/10^{5})(1+\gamma B)$	Replaces power law with exponential growth.
Formula6	$\mathrm{MS}=\exp(\alpha +\ln(\frac{{\mathrm{Mw}}^{\beta }}{{\mathrm{PDI}}^{\left|\gamma \right|}})+\delta \;\times \;B)$	Extends single-variable Mw to the combination of Mw, PDI and Br in log-space.
Formula7	MS= ${\alpha \mathrm{Mz}}^{\beta }\exp(-\gamma \cdot \mathrm{clip}(\mathrm{Mw}/\mathrm{Mz},\;\max(\mathrm{Mw}),\;0))$	Shifts from Mw to Mz, assuming high-moment species dominate MS. clip(x, a, b) = min(max(x, a), b).
Formula8	MS= ${\alpha \mathrm{Mw}}^{\beta }\;\times \;{\left(\frac{\mathrm{Mw}}{\mathrm{PDI}}\right)}^{{\gamma e}^{-\lambda \mathrm{Mw}/10^{5}}}{\;\times \;(1+\eta B)}^{\sigma }\cdot \frac{{\left(\mathrm{Mz}/\mathrm{Mw}\right)}^{\theta }}{{1+\kappa (\mathrm{Mz}/\mathrm{Mw})}^{\theta }}$	Integrates multiple nonlinear couplings (adaptive PDI effect, saturating Mz/Mw response).
Formula9	${\mathrm{MS}=\alpha \;\times \;\mathrm{HMW}}^{\beta }$	Extends MH by replace Mw with high molecular weight (HMW).
Formula10	${\mathrm{MS}=\alpha \;\times \;\exp(\ln(\mathrm{HMW}}^{\beta }+\epsilon ))\;\times \;(1+\gamma \;\times \;B)$	LCB introduces additional topological constraints to HMW region.
Formula11	${\mathrm{MS}=\exp(\alpha +\mathrm{HMW}}^{\beta }+\ln(\frac{1}{{\mathrm{PDI}}^{\left|\gamma \right|}})+\delta \;\times \;B)$	Replace Mw with HMW from Formula6.
Formula12	${\mathrm{MS}=\exp(\alpha +\mathrm{HMW}}^{\beta }+\ln(\frac{{\mathrm{Mw}}^{\eta }}{{\mathrm{PDI}}^{\left|\gamma \right|}})+\delta \;\times \;B)$	Extenson of Formula6 with HMW in log space.

**Table 3:** Performance comparison of candidate models for MS prediction across 20 independent train–test splits. Models are sorted in descending order of SCORE. Formula11, with a log-linear structure incorporating HMW, exhibits the highest overall accuracy and robustness among the physically interpretable formulas.

Model	SCORE	Test MAPE	AIC	BIC	Sphy	Training-Set R^2^	Test-Set R^2^	Test RMSE
Formula11	1.0000	1.17	−166.274	−76.305	1	0.959	0.860	0.003
Formula2	0.9963	1.08	−164.274	−73.597	1	0.959	0.833	0.003
Formula8	0.9930	1.28	−162.274	−70.889	1	0.932	0.801	0.003
Formula12	0.9924	1.04	−162.274	−70.889	1	0.966	0.822	0.003
Formula9	0.9806	1.33	−159.644	−66.574	1	0.947	0.855	0.004
Formula3	0.9759	1.06	−157.644	−74.698	1	0.962	0.798	0.004
Formula1	0.9753	1.93	−159.644	−66.574	1	0.883	0.682	0.004
Formula4	0.9747	1.31	−157.644	−71.990	1	0.936	0.759	0.004
Formula10	0.9747	1.24	−155.644	−69.282	1	0.954	0.854	0.004
Formula6	0.9733	1.07	−155.644	−69.282	1	0.961	0.808	0.004
Formula5	0.8319	4.12	−127.296	−56.816	1	0.495	−0.258	0.011
PINNs	0.7468	0.81	−8.950	123.629	1	0.974	0.712	0.005
XGBoost	0.4366	0.23	5.151	130.679	0	0.998	0.362	0.008
Formula7	0.3978	10.62	−96.214	−39.567	1	−2.367	−7.291	0.029
Random Forest	0.3752	0.08	−54.055	32.754	0	0.997	−1.947	0.016

## Data Availability

The original contributions presented in this study are included in the article/Supplementary Materials. Further inquiries can be directed to the corresponding authors.

## Supplementary Materials

The following supporting information can be downloaded at: https://www.mdpi.com/article/10.3390/polym18192421/s1, Figure S1: Correlation Analysis of MS with Various Variables; Figure S2:Relationship Between High Molecular Weight (HMW) and Polydispersity Index (PDI) Derived from Historical Production Data; Table S1: Primary Data of Melt Strength (N) and Selected GPC Parameters for LDPE 2426H. Table S2: Representative Rheotens Force-Speed Profiles for LDPE 2426H; Table S3: Train and Test R^2^ and RMSE for Break Force and FCI Predictive Models across 20 Repeated Train-test Splits; Table S4: Standard Deviations of Train-set and Test-set R^2^ and RMSE across 20 Repeated Train-test Splits for Break Force and FCI Models; Table S5: 95% Confidence Intervals of Model R2 Metrics; Table S6: 95% Confidence Intervals of Model RMSE Metrics; Table S7: Additional Test Data of Melt Strength (N) and Selected GPC Parameters for LDPE 2426H; Table S8: Additional Test Data of Melt Strength (N) and Selected GPC Parameters for LDPE 1810D.
