# Supplementary material for: An Efficient Descriptor for Tubular LDPE Melt Strength via Physics-Guided Learning and Diffusion-Based Inverse Design
Source: Polymers (Basel). 2026 Oct 4;18(19):2421. doi: 10.3390/polym18192421 (PMC13667234; doi:10.3390/polym18192421)
Supplement: Supplementary file 1 [file polymers-18-02421-s001.zip › polymers-4558026-supplementary.pdf]

# Supplementary Materials

# An Efficient Descriptor for Tubular LDPE Melt Strength via Physics-Guided Learning and Diffusion-Based Inverse Design

*Yifei Wang, Guogang Liu, Lin Liu, Yue Yu, Peiqian Yu, Linge Ma, Linlin Wei, Zhe Zhou, Linfeng Chen, Delin Yuan*, Rongjuan Cong**

National Institute of Clean-and-Low-Carbon Energy, Beijing 102209, China

*Corresponding authors:

Delin Yuan, Ph.D.

National Institute of Clean-and-Low-Carbon Energy (NICE)

Beijing 102209, China

Tel: 86-10-57337359| Email: 20027932@ceic.com

Rongjuan Cong, Ph.D.

National Institute of Clean-and-Low-Carbon Energy (NICE)

Beijing 102209, China

Tel: 86-10-57337229 | Email: 20098502@ceic.com

# Supporting Information

## Definition of GPC-Derived Molecular Features

The weight-average molecular weight (Mw), number-average molecular weight (Mn), polydispersity (PDI, also denoted as Đ, equals to Mw/Mn), z-average molecular weight (Mz), bulk intrinsic viscosity (IV), peak molecular weight (Mp), viscosity-average molecular weight (Mv), and branching index (B) are common molecular descriptors derived from triple detector gel-permeation chromatography (GPC). Dispersity PDI quantifies the breadth of molecular weight distribution. . Mp corresponds to the molecular weight value at the maximum of the backbone MWD distribution curve. Mv denotes viscosity-average molecular weight, calculated from intrinsic viscosity via the Mark–Houwink relationship.Bulk Intrinsic viscosity (IV) describes the inherent viscosity contribution of dissolved polymer chains at infinite dilution, which is strongly affected by both molecular weight magnitude and chain coil contraction induced by long chain branching. The branching index (B) is a topological descriptor used to characterize chain branching, proposed by Wallace Yau.

## Diagnostic Analysis of Melt Strength (MS) Dependencies on Key Molecular Features

To uncover the true functional relationships between polymer microstructure and macroscopic mechanical performance—and to construct physically interpretable

scaling laws—we systematically diagnosed how mechanical strength (MS) depended on five key GPC-derived descriptors: weight-average molecular weight (Mw), polydispersity index (PDI), branching index (B), z-average molecular weight (Mz), and high molecular weight tail content (HMW). To ensure fair comparison across different functional transformations and eliminate scale effects, all raw variables were subjected to a unified anonymization and normalization procedure that preserves relative rankings and local variations while preventing reverse engineering of original values. For each descriptor, we evaluated three canonical functional forms—linear (MS vs. X), logarithmic (MS vs. log X), and exponential (MS vs. exp(X))—quantified by Pearson correlation coefficients as measures of linear fit quality in the transformed space.

The results reveal:

(1) MS exhibits an exceptionally strong linear relationship with Mw ($r = 0.947$), while MS vs. exp(Mw) also achieves high correlation ($r = 0.927$); in contrast, MS vs. log(Mw) shows comparatively lower correlation ($r = 0.904$). This pattern suggests that the influence of Mw on MS lies-in the regime spanned by linear and exponential type relationships, potentially better captured by higher order power-law forms with logarithmic corrections (e.g., $MS \propto Mw \cdot \log(Mw)$ or $MS \propto Mw^n$ with $n > 1$). Accordingly, our scaling laws prioritize linear Mw terms but incorporate logarithmic or exponential augmentations to account for nonlinear reinforcement effects.

(2) HMW demonstrates near-equally strong correlations with MS under both linear (r = 0.971) and exponential (r = 0.974) transformations, indicating that the contribution of the high MW tail can be interpreted either as a direct proportional effect or as a rapidly saturating exponential response. Given their comparable performance, both linear and exponential forms of HMW are retained in subsequent models to accommodate alternative physical interpretations.

(3) The branching index B shows consistently high and similar correlations across all three transformations ($r \approx 0.90$), implying robust dependence without a clear functional preference. Consequently, multiple functional forms of B are included in the candidate model library to enhance flexibility.

(4) Mz displays only weak overall correlation with MS ($r \approx 0.6$), with no transformation yielding substantial improvement. Nevertheless, due to its distinct physical significance—particularly its sensitivity to long-chain entanglements—we retain Mz in select composite models to explore potential synergistic effects, even if its marginal predictive power is limited.

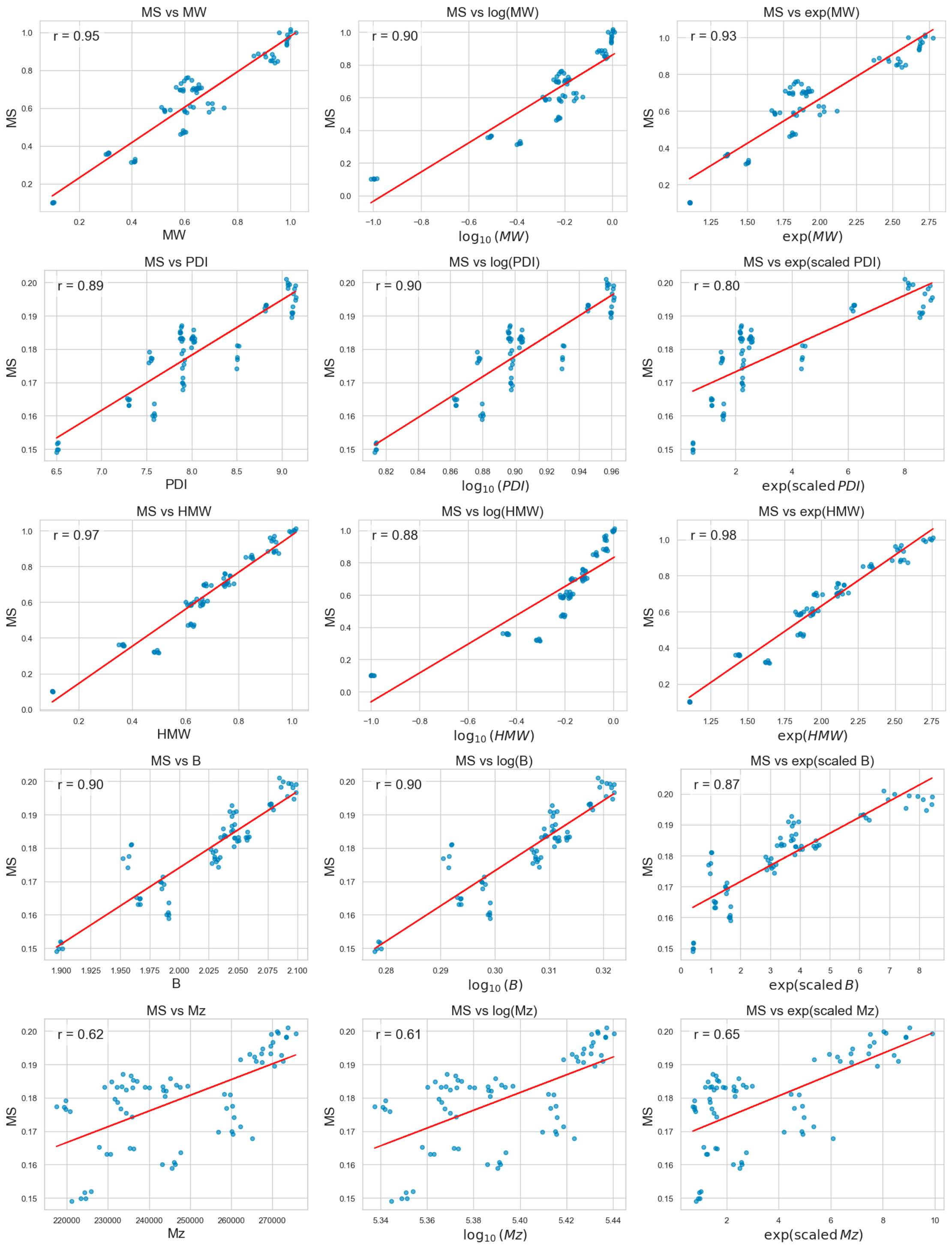


Figure S1 Correlation Analysis of MS with Various Variables. This figure illustrates the correlation coefficients (r) between MS and different variables (Mw, PDI, HMW, B, Mz) under various mathematical transformations (linear, logarithmic, exponential).

# Melt Strength and GPC Raw Data

Table S1 Primary Data of Melt Strength (N)and Selected GPC Parameters for LDPE 2426H

| ID | MS | Mw | Mn | PDI | Mz | Mv | Mp | B | HMW |
|---|---|---|---|---|---|---|---|---|---|
| 1 | 0.182 | 78600 | 15600 | 5.03 | 230800 | 66200 | 48100 | 1.276 | 0.229 |
| 2 | 0.177 | 78700 | 15800 | 4.99 | 234200 | 66300 | 48100 | 1.258 | 0.228 |
| 3 | 0.197 | 85700 | 15500 | 5.53 | 270500 | 71300 | 48100 | 1.300 | 0.246 |
| 4 | 0.177 | 77600 | 15600 | 4.97 | 219000 | 65500 | 47700 | 1.260 | 0.225 |
| 5 | 0.191 | 85100 | 15400 | 5.53 | 270800 | 70800 | 48000 | 1.268 | 0.240 |
| 6 | 0.186 | 78700 | 15600 | 5.04 | 235400 | 66200 | 48000 | 1.268 | 0.234 |
| 7 | 0.183 | 79500 | 15800 | 5.04 | 246100 | 66700 | 47500 | 1.263 | 0.235 |
| 8 | 0.199 | 86300 | 15700 | 5.5 | 275600 | 71700 | 48500 | 1.294 | 0.251 |
| 9 | 0.193 | 83500 | 15800 | 5.3 | 264000 | 69800 | 49500 | 1.289 | 0.246 |
| 10 | 0.170 | 78800 | 15500 | 5.09 | 259500 | 65700 | 46500 | 1.230 | 0.226 |
| 11 | 0.183 | 79400 | 15600 | 5.09 | 241900 | 66600 | 48000 | 1.270 | 0.235 |
| 12 | 0.164 | 72200 | 15200 | 4.74 | 232600 | 60600 | 44300 | 1.220 | 0.208 |
| 13 | 0.150 | 68000 | 15300 | 4.45 | 224100 | 57200 | 42600 | 1.176 | 0.191 |
| 14 | 0.162 | 74800 | 15000 | 4.99 | 244800 | 62400 | 44400 | 1.233 | 0.217 |
| 15 | 0.177 | 80800 | 15000 | 5.38 | 258100 | 67200 | 46400 | 1.213 | 0.227 |

Table S2 Representative Rheotens Force-Speed Profiles for LDPE 2426H

| ID | Velocity | Force | ID | Velocity | Force | ID | Velocity | Force |
|---|---|---|---|---|---|---|---|---|
| 1 | 0.7004 | 0.0001 | 67 | 1.6430 | 0.0700 | 133 | 3.8520 | 0.1270 |
| 2 | 0.7095 | 0.0008 | 68 | 1.6640 | 0.0713 | 134 | 3.9020 | 0.1275 |
| 3 | 0.7187 | 0.0015 | 69 | 1.6860 | 0.0725 | 135 | 3.9530 | 0.1281 |
| 4 | 0.7280 | 0.0022 | 70 | 1.7070 | 0.0738 | 136 | 4.0040 | 0.1286 |
| 5 | 0.7375 | 0.0029 | 71 | 1.7300 | 0.0750 | 137 | 4.0560 | 0.1291 |
| 6 | 0.7471 | 0.0036 | 72 | 1.7520 | 0.0762 | 138 | 4.1090 | 0.1297 |
| 7 | 0.7568 | 0.0043 | 73 | 1.7750 | 0.0774 | 139 | 4.1630 | 0.1302 |
| 8 | 0.7666 | 0.0051 | 74 | 1.7980 | 0.0786 | 140 | 4.2170 | 0.1307 |
| 9 | 0.7766 | 0.0058 | 75 | 1.8210 | 0.0798 | 141 | 4.2710 | 0.1312 |
| 10 | 0.7867 | 0.0066 | 76 | 1.8450 | 0.0809 | 142 | 4.3270 | 0.1317 |
| 11 | 0.7969 | 0.0074 | 77 | 1.8690 | 0.0820 | 143 | 4.3830 | 0.1321 |
| 12 | 0.8073 | 0.0082 | 78 | 1.8930 | 0.0832 | 144 | 4.4400 | 0.1326 |
| 13 | 0.8178 | 0.0090 | 79 | 1.9180 | 0.0843 | 145 | 4.4980 | 0.1331 |
| 14 | 0.8284 | 0.0098 | 80 | 1.9430 | 0.0854 | 146 | 4.5560 | 0.1336 |
| 15 | 0.8392 | 0.0106 | 81 | 1.9680 | 0.0864 | 147 | 4.6160 | 0.1340 |
| 16 | 0.8501 | 0.0114 | 82 | 1.9940 | 0.0875 | 148 | 4.6760 | 0.1345 |
| 17 | 0.8611 | 0.0122 | 83 | 2.0200 | 0.0886 | 149 | 4.7360 | 0.1349 |
| 18 | 0.8723 | 0.0131 | 84 | 2.0460 | 0.0896 | 150 | 4.7980 | 0.1354 |
| 19 | 0.8837 | 0.0139 | 85 | 2.0720 | 0.0906 | 151 | 4.8600 | 0.1358 |
| 20 | 0.8952 | 0.0148 | 86 | 2.0990 | 0.0916 | 152 | 4.9230 | 0.1362 |
| 21 | 0.9068 | 0.0157 | 87 | 2.1270 | 0.0926 | 153 | 4.9870 | 0.1367 |
| 22 | 0.9186 | 0.0166 | 88 | 2.1540 | 0.0936 | 154 | 5.0520 | 0.1371 |

| | | | | | | | | |
|---|---|---|---|---|---|---|---|---|
| 23 | 0.9305 | 0.0175 | 89 | 2.1820 | 0.0946 | 155 | 5.1180 | 0.1375 |
| 24 | 0.9426 | 0.0184 | 90 | 2.2110 | 0.0955 | 156 | 5.1850 | 0.1379 |
| 25 | 0.9549 | 0.0193 | 91 | 2.2390 | 0.0965 | 157 | 5.2520 | 0.1383 |
| 26 | 0.9673 | 0.0203 | 92 | 2.2680 | 0.0974 | 158 | 5.3200 | 0.1387 |
| 27 | 0.9798 | 0.0212 | 93 | 2.2980 | 0.0983 | 159 | 5.3890 | 0.1391 |
| 28 | 0.9926 | 0.0222 | 94 | 2.3280 | 0.0993 | 160 | 5.4590 | 0.1395 |
| 29 | 1.0050 | 0.0232 | 95 | 2.3580 | 0.1002 | 161 | 5.5300 | 0.1399 |
| 30 | 1.0190 | 0.0242 | 96 | 2.3890 | 0.1010 | 162 | 5.6020 | 0.1402 |
| 31 | 1.0320 | 0.0252 | 97 | 2.4200 | 0.1019 | 163 | 5.6750 | 0.1406 |
| 32 | 1.0450 | 0.0262 | 98 | 2.4510 | 0.1028 | 164 | 5.7490 | 0.1410 |
| 33 | 1.0590 | 0.0272 | 99 | 2.4830 | 0.1036 | 165 | 5.8240 | 0.1414 |
| 34 | 1.0730 | 0.0282 | 100 | 2.5150 | 0.1045 | 166 | 5.8990 | 0.1417 |
| 35 | 1.0870 | 0.0293 | 101 | 2.5480 | 0.1053 | 167 | 5.9760 | 0.1421 |
| 36 | 1.1010 | 0.0304 | 102 | 2.5810 | 0.1061 | 168 | 6.0540 | 0.1424 |
| 37 | 1.1150 | 0.0314 | 103 | 2.6150 | 0.1070 | 169 | 6.1320 | 0.1428 |
| 38 | 1.1290 | 0.0325 | 104 | 2.6490 | 0.1078 | 170 | 6.2120 | 0.1431 |
| 39 | 1.1440 | 0.0337 | 105 | 2.6830 | 0.1085 | 171 | 6.2930 | 0.1434 |
| 40 | 1.1590 | 0.0348 | 106 | 2.7180 | 0.1093 | 172 | 6.3750 | 0.1438 |
| 41 | 1.1740 | 0.0359 | 107 | 2.7530 | 0.1101 | 173 | 6.4570 | 0.1441 |
| 42 | 1.1890 | 0.0371 | 108 | 2.7890 | 0.1109 | 174 | 6.5410 | 0.1444 |
| 43 | 1.2050 | 0.0382 | 109 | 2.8250 | 0.1116 | 175 | 6.6260 | 0.1447 |
| 44 | 1.2200 | 0.0394 | 110 | 2.8620 | 0.1123 | 176 | 6.7130 | 0.1451 |
| 45 | 1.2360 | 0.0406 | 111 | 2.8990 | 0.1131 | 177 | 6.8000 | 0.1454 |
| 46 | 1.2520 | 0.0419 | 112 | 2.9370 | 0.1138 | 178 | 6.8880 | 0.1457 |
| 47 | 1.2690 | 0.0431 | 113 | 2.9750 | 0.1145 | 179 | 6.9780 | 0.1460 |
| 48 | 1.2850 | 0.0443 | 114 | 3.0140 | 0.1152 | 180 | 7.0680 | 0.1463 |
| 49 | 1.3020 | 0.0456 | 115 | 3.0530 | 0.1159 | 181 | 7.1600 | 0.1466 |
| 50 | 1.3190 | 0.0469 | 116 | 3.0930 | 0.1166 | 182 | 7.2530 | 0.1469 |
| 51 | 1.3360 | 0.0482 | 117 | 3.1330 | 0.1173 | 183 | 7.3480 | 0.1472 |
| 52 | 1.3530 | 0.0495 | 118 | 3.1740 | 0.1179 | 184 | 7.4430 | 0.1475 |
| 53 | 1.3710 | 0.0508 | 119 | 3.2150 | 0.1186 | 185 | 7.5400 | 0.1477 |
| 54 | 1.3890 | 0.0522 | 120 | 3.2570 | 0.1193 | 186 | 7.6380 | 0.1480 |
| 55 | 1.4070 | 0.0535 | 121 | 3.2990 | 0.1199 | 187 | 7.7370 | 0.1483 |
| 56 | 1.4250 | 0.0549 | 122 | 3.3420 | 0.1205 | 188 | 7.8380 | 0.1486 |
| 57 | 1.4440 | 0.0563 | 123 | 3.3850 | 0.1212 | 189 | 7.9400 | 0.1488 |
| 58 | 1.4620 | 0.0578 | 124 | 3.4290 | 0.1218 | 190 | 8.0430 | 0.1491 |
| 59 | 1.4810 | 0.0592 | 125 | 3.4740 | 0.1224 | 191 | 8.1470 | 0.1493 |
| 60 | 1.5010 | 0.0606 | 126 | 3.5190 | 0.1230 | 192 | 8.2530 | 0.1496 |
| 61 | 1.5200 | 0.0620 | 127 | 3.5650 | 0.1236 | 193 | 8.3610 | 0.1499 |
| 62 | 1.5400 | 0.0634 | 128 | 3.6110 | 0.1242 | 194 | 8.4690 | 0.1501 |
| 63 | 1.5600 | 0.0647 | 129 | 3.6580 | 0.1248 | 195 | 8.5790 | 0.1504 |
| 64 | 1.5800 | 0.0661 | 130 | 3.7060 | 0.1253 | 196 | 8.6910 | 0.1506 |
| 65 | 1.6010 | 0.0674 | 131 | 3.7540 | 0.1259 | 197 | 8.8040 | 0.1508 |

| | | | | | | | | |
|---|---|---|---|---|---|---|---|---|
| 66 | 1.6210 | 0.0687 | 132 | 3.8030 | 0.1265 | 198 | 9.0340 | 0.1513 |

## Performance of Predictive Modeling with Break Force and FCI

Modelling and testing were performed following the same workflow adopted in the main manuscript. Although FCI and Break Force exhibited strong linear correlations with GPC features in correlation analysis, the overall model performance in terms of train and test $R^2$ and RMSE were inferior to that obtained using the composite surrogate MS (Table S3 and S4). While the training performance of model with Break Force is reasonable, the test performance is poor, indicating limited generalisation capability. Furthermore, the substantial variance observed in both train and test $R^2$ and RMSE further suggests that predictions built upon these parameters have limited reliability. In addition, when modelling with FCI and Break Force, Formula 11 still achieves competitive predictive performance and ranks among the top three models.

Table S3 Train and Test $R^2$ and RMSE for Break Force and FCI Predictive Models across 20 Repeated Train-test Splits.

| Model | FCI | | | | Break Force | | | |
|---|---|---|---|---|---|---|---|---|
| | Train $R^2$ | Test $R^2$ | Train RMSE | Test RMSE | Train $R^2$ | Test $R^2$ | Train RMSE | Test RMSE |
| Formula6 | 0.246 | -3.923 | 22.043 | 23.624 | 0.925 | 0.244 | 0.003 | 0.005 |
| Formula12 | 0.248 | -4.280 | 22.018 | 24.039 | 0.924 | 0.220 | 0.003 | 0.005 |
| Formula11 | 0.251 | -4.349 | 21.983 | 23.702 | 0.838 | -0.27 | 0.005 | 0.005 |
| Formula4 | 0.186 | -5.344 | 22.921 | 30.170 | 0.928 | 0.118 | 0.003 | 0.005 |
| Formula3 | -0.258 | -5.609 | 28.222 | 29.406 | 0.930 | 0.020 | 0.003 | 0.005 |
| PINNs | 0.814 | -6.018 | 10.972 | 27.209 | 0.938 | -0.61 | 0.003 | 0.008 |
| XGBoost | 1.000 | -8.916 | 0.001 | 27.526 | 0.997 | -1.51 | 0.001 | 0.009 |
| Formula8 | -12.10 | -136.7 | 91.332 | 103.69 | 0.923 | 0.285 | 0.003 | 0.004 |
| Random Forest | -10.29 | -635.1 | 82.626 | 211.22 | 0.992 | -5.49 | 0.001 | 0.020 |
| Formula5 | -229.4 | -2407 | 384.60 | 390.19 | 0.403 | -2.23 | 0.009 | 0.010 |
| Formula2 | -375.5 | -3883 | 491.73 | 496.88 | 0.927 | 0.204 | 0.003 | 0.005 |
| Formula10 | -392.4 | -4053 | 502.63 | 507.74 | 0.840 | -0.200 | 0.005 | 0.005 |
| Formula7 | -538.6 | -5515 | 588.70 | 593.15 | -1.99 | -13.4 | 0.021 | 0.026 |

| | | | | | | | | |
|---|---|---|---|---|---|---|---|---|
| Formula1 | -538.9 | -5521 | 588.85 | 593.36 | 0.847 | 0.024 | 0.005 | 0.004 |
| Formula9 | -540.9 | -5542 | 589.99 | 594.50 | 0.839 | -0.19 | 0.005 | 0.005 |

Table S4 Standard Deviations of Train-set and Test-set $R^2$ and RMSE across 20 Repeated Train-test Splits for Break Force and FCI Models.

| Model | FCI | | Break Force | | MS | |
|---|---|---|---|---|---|---|
| | Train $R^2$_std | Test $R^2$_std | Train $R^2$_std | Test $R^2$_std | Train $R^2$_std | Test $R^2$_std |
| Formula10 | 0.0479 | 3.8173 | 0.0479 | 3.8173 | 0.0083 | 0.1541 |
| Formula9 | 0.0483 | 3.8167 | 0.0483 | 3.8167 | 0.0110 | 0.1565 |
| Formula11 | 0.0492 | 4.1174 | 0.0492 | 4.1174 | 0.0093 | 0.1586 |
| Formula2 | 0.0321 | 1.7448 | 0.0321 | 1.7448 | 0.0127 | 0.1853 |
| Formula12 | 0.0328 | 1.7811 | 0.0328 | 1.7811 | 0.0098 | 0.1917 |
| Formula3 | 0.0328 | 2.1675 | 0.0328 | 2.1675 | 0.0118 | 0.2127 |
| Formula6 | 0.0324 | 1.7492 | 0.0324 | 1.7492 | 0.0110 | 0.2171 |
| Formula8 | 0.0321 | 1.7355 | 0.0321 | 1.7355 | 0.0289 | 0.2604 |
| Formula4 | 0.0324 | 2.0437 | 0.0324 | 2.0437 | 0.0283 | 0.2627 |
| PINNs | 0.0156 | 4.6374 | 0.0156 | 4.6374 | 0.0106 | 0.2829 |
| Formula1 | 0.0411 | 3.3626 | 0.0411 | 3.3626 | 0.0357 | 0.4599 |
| XGBoost | 0.0011 | 7.8893 | 0.0011 | 7.8893 | 0.0006 | 0.5467 |
| Formula5 | 0.0311 | 9.9859 | 0.0311 | 9.9859 | 0.0223 | 1.3748 |
| Formula7 | 0.3846 | 37.2645 | 0.3846 | 37.2645 | 0.3125 | 7.9707 |
| Random Forest | 0.0230 | 11.1165 | 0.0230 | 11.1165 | 0.0035 | 19.5301 |

## Confidence Intervals (CI) for Model Performance Metrics

The 95% confidence interval for the mean of test-set metrics ($R^2$ and RMSE) over 20 repeated group-aware train–test splits was calculated. Each model collected 20 metric values from repeated partitioning. The sample standard deviation (s) of these 20 values was computed. The standard error of the mean was obtained as $SE = s/\sqrt{n}$, where n=20. The 95% confidence interval was then calculated using the t-distribution. The equation to calculate CI is:

$$CI = \bar{x} \pm 2.093 \cdot s/\sqrt{n}$$

where $\bar{x}$ denoted the arithmetic mean of the 20 metric values.

As Table S5 and S6 show. the 95% train and test confidence intervals of Formula11 were the lowest, accompanied by relatively narrow confidence intervals for train and test R2, further demonstrating the reliable predictive capacity of this model.

Table S5 95% Confidence Intervals of Model $R^2$ Metrics.

| Model | train_$R^2$_ci_low | train_$R^2$_ci_high | train_$R^2$_ci_width | test_$R^2$_ci_low | test_$R^2$_ci_high | test_$R^2$_ci_width |
|---|---|---|---|---|---|---|
| Formula11 | 0.9550 | 0.9639 | 0.0089 | 0.7835 | 0.9358 | 0.1523 |
| Formula10 | 0.9504 | 0.9584 | 0.0080 | 0.7795 | 0.9275 | 0.1479 |
| Formula9 | 0.9417 | 0.9522 | 0.0106 | 0.7793 | 0.9297 | 0.1503 |
| Formula2 | 0.9527 | 0.9649 | 0.0122 | 0.7442 | 0.9222 | 0.1780 |
| Formula12 | 0.9610 | 0.9704 | 0.0094 | 0.7297 | 0.9138 | 0.1841 |
| Formula6 | 0.9561 | 0.9667 | 0.0106 | 0.7037 | 0.9122 | 0.2085 |
| Formula3 | 0.9561 | 0.9674 | 0.0113 | 0.6959 | 0.9002 | 0.2043 |
| Formula8 | 0.9181 | 0.9459 | 0.0278 | 0.6764 | 0.9265 | 0.2501 |
| Formula4 | 0.9222 | 0.9494 | 0.0272 | 0.6328 | 0.8851 | 0.2523 |
| PINNs | 0.9684 | 0.9786 | 0.0102 | 0.5761 | 0.8478 | 0.2717 |
| Formula1 | 0.8658 | 0.9001 | 0.0343 | 0.4614 | 0.9031 | 0.4417 |
| XGBoost | 0.9973 | 0.9978 | 0.0006 | 0.0996 | 0.6247 | 0.5251 |
| Formula5 | 0.4843 | 0.5057 | 0.0214 | -0.9185 | 0.4018 | 1.3203 |
| Random Forest | 0.9941 | 1.0000 | 0.0059 | -6.6632 | -0.7053 | 5.9579 |
| Formula7 | -2.5172 | -2.2171 | 0.3001 | -11.1180 | -3.4633 | 7.6547 |

Table S6 95% Confidence Intervals of Model RMSE Metrics

| Model | Train RMSE_ci_low | Train RMSE_ci_high | Train RMSE_ci_width | Test RMSE_ci_low | Test RMSE_ci_high | Test RMSE_ci_width |
|---|---|---|---|---|---|---|
| Formula11 | 0.0024 | 0.0026 | 0.0002 | 0.0027 | 0.0039 | 0.0012 |
| Formula10 | 0.0025 | 0.0028 | 0.0003 | 0.0028 | 0.0042 | 0.0014 |
| Formula9 | 0.0027 | 0.0030 | 0.0003 | 0.0028 | 0.0042 | 0.0014 |
| Formula2 | 0.0024 | 0.0026 | 0.0003 | 0.0025 | 0.0040 | 0.0015 |
| Formula12 | 0.0022 | 0.0024 | 0.0003 | 0.0028 | 0.0041 | 0.0013 |
| Formula6 | 0.0023 | 0.0026 | 0.0003 | 0.0028 | 0.0044 | 0.0016 |
| Formula3 | 0.0023 | 0.0025 | 0.0003 | 0.0029 | 0.0044 | 0.0015 |

| | | | | | | |
|---|---|---|---|---|---|---|
| Formula8 | 0.0030 | 0.0034 | 0.0004 | 0.0026 | 0.0041 | 0.0014 |
| Formula4 | 0.0029 | 0.0033 | 0.0004 | 0.0029 | 0.0046 | 0.0017 |
| PINNs | 0.0018 | 0.0023 | 0.0005 | 0.0037 | 0.0073 | 0.0036 |
| Formula1 | 0.0041 | 0.0043 | 0.0003 | 0.0036 | 0.0049 | 0.0013 |
| XGBoost | 0.0006 | 0.0006 | 4.5e-05 | 0.0070 | 0.0088 | 0.0019 |
| Formula5 | 0.0085 | 0.0094 | 0.0009 | 0.0093 | 0.0119 | 0.0026 |
| Random Forest | 9.1e-05 | 0.0005 | 0.0004 | 0.0130 | 0.0330 | 0.0200 |
| Formula7 | 0.0219 | 0.0243 | 0.0023 | 0.0246 | 0.0335 | 0.0089 |

## Model Transfer method via Constrained Refinement

To extend the predictive capability of the originally developed empirical model (termed Formula11)—trained on melt strength (MS) data for LDPE grade 2426H —to LDPE grade 1810D, a minimal-parameter adaptation strategy was adopted which preserved the physical interpretability and sign constraints of the original formulation. The base model expressed the melt strength as:

$$MS=exp(\alpha+HMW^{\beta}+ln(\frac{1}{PDI^{|\gamma|}})+\delta\times B)$$

The original parameters (α, β, γ, δ) were derived from LDPE 2426H data under the physically motivated constraints $\beta>0$, $\gamma<0$, and $\delta>0$, reflecting expected trends in MS with respect to chain length, breadth of molecular weight distribution, and thermal effects.

Although the original model—trained on LDPE grade 2426H—already yielded reasonable predictive accuracy on LDPE 1810D without any adaptation (suggesting a degree of inherent transferability across polyethylene architectures), a further action was sought to quantify the extent of systematic parameter shift required to align the model with LDPE specific rheological behavior. Given the limited availability of

LDPE data and the risk of overfitting, we refrained from full re-optimization. Instead, sequential single-parameter refinement was performed: each parameter was individually re-estimated via ordinary least squares in log-space while fixing the others at their original values, subject to maintaining its prescribed physical sign. Specifically:

$\alpha$ was updated to the mean residual offset

$$\alpha^{*}=\ln(MS_{true})-(\beta\times HMW+\gamma\ln(PDI)+\delta\times B)$$

$\beta$, $\gamma$ and $\delta$ were updated via linear regression of corresponding covariate against the partial residual:

$$\beta^{*}=\frac{\sum(HMW)_{i}\cdot r_{i}}{\sum(HMW)_{i}^{2}}$$

Where $r_i=\ln(MS_{true})-\alpha-\gamma\ln(PDI)_i+\delta\times B_i$

Only updates that preserved the original sign constraints were accepted. Among all candidate refinements, the simultaneous adjustment of the intercept α and the temperature coefficient δ yielded the largest reduction in log-mean-squared error, while maintaining $\alpha < 0$ and $\delta > 0$—fully consistent with the model's physical assumptions. This two-parameter refined set was therefore adopted for final prediction on the 1810D dataset.

This approach ensures minimal deviation from the original Physics-guided structure, avoids over parameterization, and enables robust transfer across polymer grades with distinct branching architectures.

Table S7 Additional Test Data of Melt Strength (N) and Selected GPC Parameters for LDPE 2426H

| ID | MS | Mw | Mn | PDI | Mz | Mv | Mp | B |
|---|---|---|---|---|---|---|---|---|

| | | | | | | | | |
|---|---|---|---|---|---|---|---|---|
| 1 | 0.201 | 75300 | 15100 | 4.97 | 216700 | 63900 | 50200 | 1.23 |
| 2 | 0.176 | 80500 | 11200 | 7.16 | 403000 | 62200 | 34000 | 1.76 |
| 3 | 0.171 | 72500 | 12800 | 5.65 | 257300 | 59400 | 40700 | 1.55 |
| 4 | 0.179 | 77500 | 15200 | 5.10 | 240400 | 65100 | 53100 | 1.25 |
| 5 | 0.189 | 79100 | 15000 | 5.29 | 265000 | 65900 | 52100 | 1.36 |

Table S8 Additional Test Data of Melt Strength (N) and Selected GPC Parameters for LDPE 1810D

| ID | MS | Mw | Mn | PDI | Mz | Mv | Mp | B |
|---|---|---|---|---|---|---|---|---|
| 1 | 0.2667 | 114600 | 16900 | 6.79 | 369800 | 93600 | 55600 | 1.71 |
| 2 | 0.290 | 115600 | 17200 | 6.74 | 374000 | 94400 | 55900 | 1.73 |
| 3 | 0.307 | 117700 | 17500 | 6.73 | 385900 | 96100 | 55200 | 1.87 |
| 4 | 0.310 | 111100 | 15900 | 6.97 | 340300 | 91200 | 55300 | 1.57 |

## Necessity of Conditional Constraints in Inverse Design

In LDPE systems, the polydispersity index (PDI) does not vary independently but is indirectly governed by the high molecular weight tail content (HMW)—a dominant structural driver. This arises from the intrinsic coupling among molecular descriptors: HMW is strongly correlated with weight-average molecular weight (Mw), and PDI itself is inherently linked to Mw through shared polymerization kinetics (e.g., chain transfer and termination rates). Consequently, PDI exhibits a secondary dependence on HMW, mediated via its mutual relationship with Mw. This hierarchical dependency implies that HMW serves not only as a direct predictor of mechanical strength but also as an implicit regulator of molecular heterogeneity (PDI). Ignoring this embedded correlation risks over-parameterizing inverse models with redundant or physically inconsistent degrees of freedom.

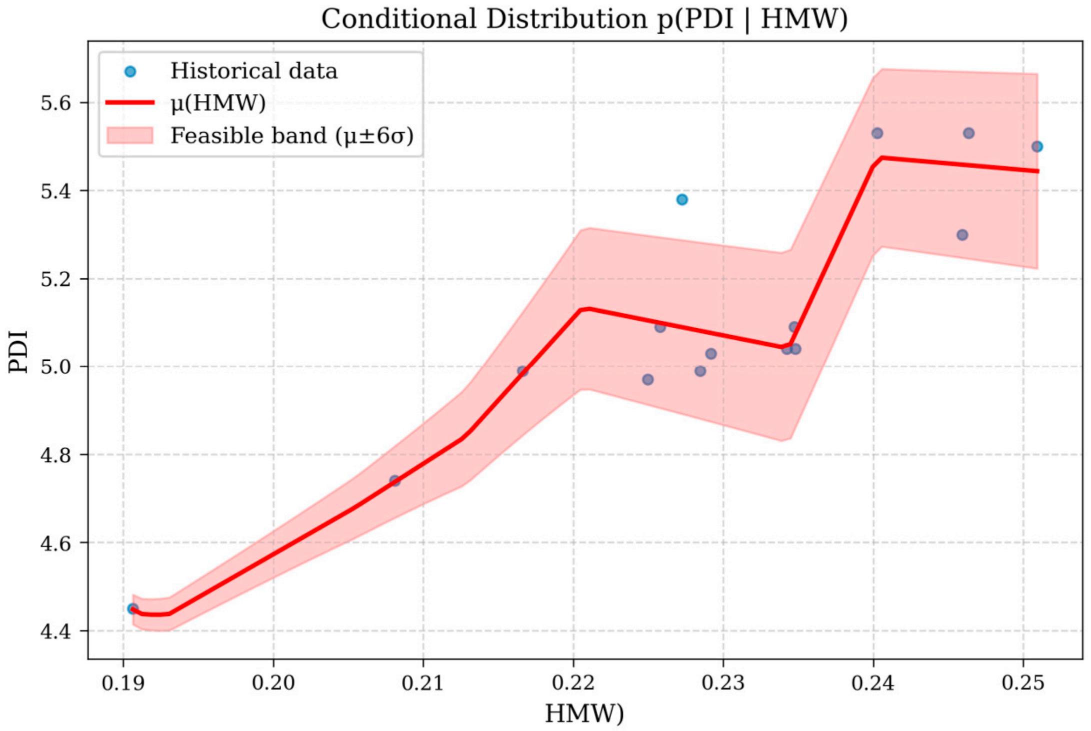


Figure S2 Relationship Between High Molecular Weight (HMW) and Polydispersity Index (PDI) Derived from Historical Production Data. The solid red line represents the mean trend, μ(HMW), while the shaded pink region denotes the μ±6σ confidence band, capturing approximately most of observed values, which defines the feasible design space for HMW-PDI combinations.
